# Tailored Immunogens Direct Affinity Maturation toward HIV Neutralizing Antibodies

**DOI:** 10.1016/j.cell.2016.08.005

**Published:** 2016-09-08

**Authors:** Bryan Briney, Devin Sok, Joseph G. Jardine, Daniel W. Kulp, Patrick Skog, Sergey Menis, Ronald Jacak, Oleksandr Kalyuzhniy, Natalia de Val, Fabian Sesterhenn, Khoa M. Le, Alejandra Ramos, Meaghan Jones, Karen L. Saye-Francisco, Tanya R. Blane, Skye Spencer, Erik Georgeson, Xiaozhen Hu, Gabriel Ozorowski, Yumiko Adachi, Michael Kubitz, Anita Sarkar, Ian A. Wilson, Andrew B. Ward, David Nemazee, Dennis R. Burton, William R. Schief

**Affiliations:** 1 Department of Immunology and Microbial Science, The Scripps Research Institute, La Jolla, CA 92037, USA; 2 IAVI Neutralizing Antibody Center, The Scripps Research Institute, La Jolla, CA 92037, USA; 3 Center for HIV/AIDS Vaccine Immunology and Immunogen Discovery, The Scripps Research Institute, La Jolla, CA 92037, USA; 4 Department of Integrative Structural and Computational Biology, The Scripps Research Institute, La Jolla, CA 92037, USA; 5 Skaggs Institute for Chemical Biology, The Scripps Research Institute, La Jolla, CA 92037, USA; 6 Ragon Institute of MGH, MIT and Harvard, Cambridge, MA 02129, USA

## Abstract

Induction of broadly neutralizing antibodies (bnAbs) is a primary goal of HIV vaccine development. VRC01-class bnAbs are important vaccine leads because their precursor B cells targeted by an engineered priming immunogen are relatively common among humans. This priming immunogen has demonstrated the ability to initiate a bnAb response in animal models, but recall and maturation toward bnAb development has not been shown. Here, we report the development of boosting immunogens designed to guide the genetic and functional maturation of previously primed VRC01-class precursors. Boosting a transgenic mouse model expressing germline VRC01 heavy chains produced broad neutralization of near-native isolates (N276A) and weak neutralization of fully native HIV. Functional and genetic characteristics indicate that the boosted mAbs are consistent with partially mature VRC01-class antibodies and place them on a maturation trajectory that leads toward mature VRC01-class bnAbs. The results show how reductionist sequential immunization can guide maturation of HIV bnAb responses.

## Highlights

• Designed boosting immunogens to follow a VRC01-class germline-targeting prime
• Boosted Abs selectively incorporate VRC01-class somatic mutations
• Sequential boosts elicit VRC01-class mAbs with broad neutralization of N276A viruses
• Vaccine-elicited VRC01-class mAbs neutralize one fully native HIV isolate

## Graphical Abstract

**Figure:**
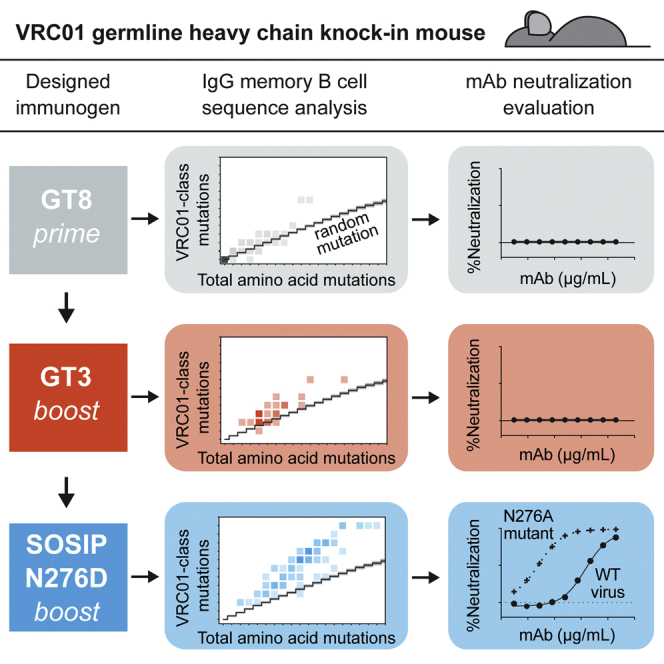

## Teaser

Development of boosting HIV envelope immunogens designed to guide the genetic and functional maturation of VRC01-class bnAbs provides a roadmap for the design of an effective HIV vaccine.

## Introduction

Elicitation of a broadly neutralizing antibody (bnAb) response is thought to be a highly desirable feature of an effective HIV vaccine (Burton et al., 2005, Pantaleo and Koup, 2004). Passive transfer of bnAbs to non-human primates (NHPs) can provide sterilizing protection against challenge by chimeric simian/human immunodeficiency viruses (SHIVs). Thus, it is widely expected that vaccine induction of sustained titers of potent bnAbs would protect humans against diverse HIV strains (Burton and Hangartner, 2016, Mascola and Haynes, 2013).

Several major challenges must be overcome to meet this goal. First, potent bnAbs targeting relatively conserved epitopes on the HIV envelope (Env) trimer develop in only a minority of infected individuals, whereas narrowly neutralizing Abs targeting variable epitopes on the infecting virus develop in all or most cases of infection (Richman et al., 2003, Wei et al., 2003). Furthermore, bnAbs have not been induced by vaccination in humans or standard animal models, while strain-specific neutralizing antibodies have been induced by vaccination with native-like trimers (Hessell et al., 2016, Sanders et al., 2015). Thus, elicitation of bnAbs will likely require strategies to focus responses to relatively conserved epitopes and may also require suppression of responses to variable epitopes. Second, bnAb inferred-germline precursors are generally not broadly cross-reactive to wild-type Env proteins: such precursors typically show no detectable affinity for wild-type Env proteins tested (Hoot et al., 2013, Jardine et al., 2013, McGuire et al., 2013, Pancera et al., 2010, Scheid et al., 2011, Xiao et al., 2009, Zhou et al., 2010) or bind to one or a limited number of Env proteins (Andrabi et al., 2015, Bhiman et al., 2015, Doria-Rose et al., 2014, Gorman et al., 2016, Haynes et al., 2012, Liao et al., 2013). Thus, elicitation of responses similar to known bnAbs will likely require “germline-targeting” priming immunogens specifically designed or selected for their ability to activate the appropriate precursor B cells (Andrabi et al., 2015, Bhiman et al., 2015, Dimitrov, 2010, Doria-Rose et al., 2014, Gorman et al., 2016, Haynes et al., 2012, Jardine et al., 2013, Jardine et al., 2015, Jardine et al., 2016a, Liao et al., 2013, Ma et al., 2011, McGuire et al., 2013, McGuire et al., 2014, McGuire et al., 2016, Pancera et al., 2010, Xiao et al., 2009, Zhou et al., 2010). Third, although HIV bnAbs target several different epitopes and use a variety of germline segments, all known bnAbs share one or more unusual genetic features: very long heavy-chain CDR3 loops; short light-chain CDR3 loops; and a high level of somatic hypermutation (SHM), including SHM-associated insertions or deletions (indels) (Doria-Rose et al., 2014, Falkowska et al., 2014, Kepler et al., 2014, Liao et al., 2013, Pancera et al., 2014, Scharf et al., 2014, Scheid et al., 2011, Sok et al., 2014, Walker et al., 2009, Walker et al., 2011, Wu et al., 2010). The high level of SHM in most bnAbs indicates a protracted maturation process in which memory B cells undergo multiple rounds of stimulation and affinity maturation driven by constantly mutating Env. Induction of highly mutated, broadly reactive antibodies by vaccination will likely require a multi-step immunization strategy in which successive distinct boosting immunogens follow a germline-targeting prime to drive antibody maturation toward a broadly neutralizing phenotype (Dimitrov, 2010, Haynes et al., 2012, Jardine et al., 2013, Jardine et al., 2015, Jardine et al., 2016b, McGuire et al., 2013, Pancera et al., 2010, Zhou et al., 2010).

VRC01-class bnAbs targeting the CD4 binding site (CD4bs) on HIV Env are attractive leads for HIV vaccine design, as they are among the most broad and potently neutralizing (Mascola and Haynes, 2013, Scheid et al., 2011, Wu et al., 2010, Wu et al., 2011) and they can protect against infection in NHPs (Pegu et al., 2014, Rudicell et al., 2014, Shingai et al., 2013) and suppress viremia in NHPs (Barouch et al., 2013, Shingai et al., 2013) and humans (Caskey et al., 2015). VRC01-class bnAbs derive from VH1-2 alleles present in ∼96% of humans and encode an unusually short (five amino acid) light-chain CDR3 loop, providing a well-defined germline target for an engineered priming immunogen (Jardine et al., 2013, McGuire et al., 2013). One such immunogen, eOD-GT8 60-mer, has been shown to target true human naive VRC01-class precursors at a frequency of 1 in 400,000 to 2.4 million naive human B cells, corresponding to 15 to 90 precursors per resting human lymph node (Jardine et al., 2016a). Thus VRC01-class bnAb precursors are abundant in humans, and we have at least one immunogen to target them.

We recently demonstrated that eOD-GT8 60-mer immunization primes relatively rare VRC01-class precursors in a transgenic mouse model expressing the VRC01 germline-reverted heavy chain (VRC01 gH) (Jardine et al., 2015). In this heterozygous knockin mouse model, ∼85% of the B cells express the VRC01 gH chain paired with diverse mouse light chains, while ∼15% of the B cells express diverse mouse heavy and light chains. Owing largely to the low frequency of mouse light chains with a 5AA CDR3 loop (∼0.1%), the frequency of VRC01-class precursors in the VRC01 gH mouse is estimated to be only ∼5-fold higher than in humans. Furthermore, in contrast to homozygous knockin mice, the VRC01 gH mouse imposes competition from diverse mouse B cell specificities that is reduced by a factor of only seven relative to a normal mouse. Despite these challenges, we found that a single immunization of eOD-GT8 60-mer in the VRC01 gH mouse resulted in activation of B cells encoding antibodies with VRC01-class genetic features, induction of specific somatic mutations shared with mature VRC01-class bnAbs, and production of mutated VRC01-class memory B cells with at least weak affinity for potential boost immunogens. As expected, although a subset of GT8-specific monoclonal antibodies (mAbs) isolated from memory-phenotype B cells in immunized VRC01 gH mice showed weak cross-reactivity to near-native Env, none of the mAbs acquired neutralizing activity (Jardine et al., 2015). The VRC01 gH mouse is thus an attractive model to evaluate boosting strategies to induce VRC01-class bnAbs following an eOD-GT8 60-mer prime, and the results in this mouse should have potential relevance to human vaccination.

Based on structure-function studies of VRC01-class bnAbs (Diskin et al., 2011, Diskin et al., 2013, Georgiev et al., 2014, Jardine et al., 2016b, Lyumkis et al., 2013, West et al., 2012, Zhou et al., 2010, Zhou et al., 2013), we have formulated a working-concept sequential immunization strategy for how to induce VRC01-class bnAbs (Jardine et al., 2016b). The strategy proposes a sequence of four types of immunogens, each with specific objectives for affinity maturation: (1) germline-targeting nanoparticles, to activate VRC01-class germline precursors and select sufficient VRC01-class mutations for low-affinity recognition of N276(−) Env lacking the N276 glycosylation site; (2) native-like N276(−) trimers, to select mutations that enable neutralization of N276(−) viruses; (3) native-like N276(+) trimers produced in GnTI^−/−^ cells (or insect cells) to present oligomannose glycans, for selection of light-chain CDR1 mutations and/or deletions to accommodate the base of the N276 glycan and to allow neutralization of viruses passaged in GnTI^−/−^ cells; and (4) native-like trimers bearing native glycans, for selection of light-chain FW3 mutations to accommodate the distal portions of the N276 glycan and to allow neutralization of viruses bearing native glycans.

In the present work, we focused on the first two steps of this strategy. We hypothesized that the eOD-GT8 60-mer prime might fail to generate memory B cells capable of being activated by native-like N276(−) trimers. Supporting this hypothesis, mAbs induced by eOD-GT8 60-mer in the VRC01 gH mouse showed affinity for an N276(−) core gp120 (Jardine et al., 2015) but not for native-like N276(−) trimers (not shown). Thus, we sought to develop immunogens that could serve as a bridge between the eOD-GT8 60-mer prime and near-native N276(−) trimers.

We report the development and testing of two such boosting immunogens, BG505 core-GT3 nanoparticle (NP) and BG505 SOSIP-GT3 trimer. We show that sequential immunization schemes employing these bridging boost immunogens drove the maturation of eOD-GT8 60-mer primed B cells toward VRC01-class bnAbs and induced broad neutralization of near-native (N276A) viruses and weak neutralization of a fully native virus in VRC01 gH mice. The results demonstrate that reductionist sequential immunization can initiate and guide maturation of pre-defined neutralizing antibody specificities. Furthermore, our findings provide a foundation on which to develop a vaccine to induce VRC01-class bnAbs.

## Results

### Engineering a Boosting Immunogen to Follow eOD-GT8 60-mer Priming of VRC01-Class Abs

**Figure 1:**
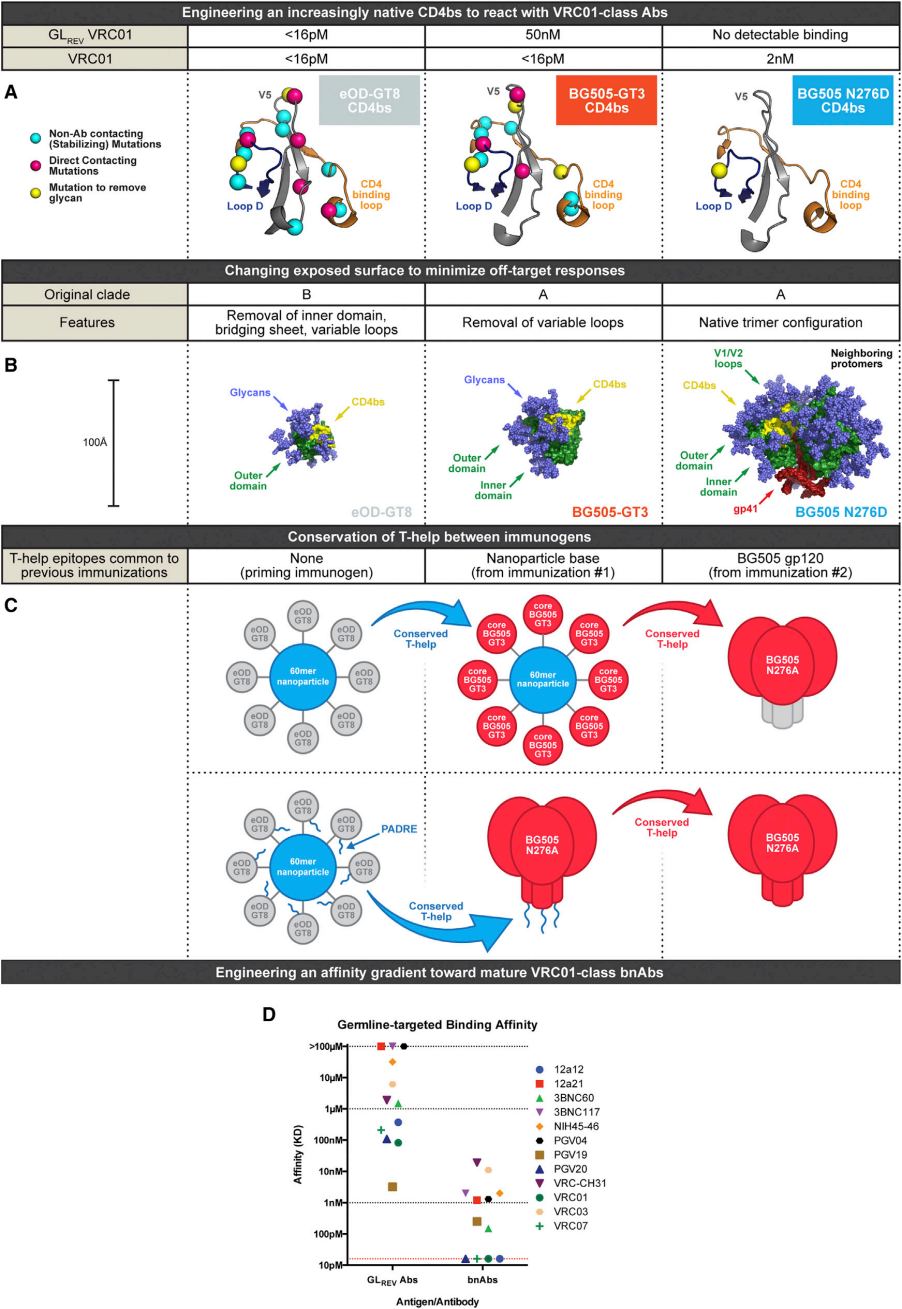
Design of a Sequential Immunization Strategy Employing BG505 GT3 (A) Design of boosting immunogens (BG505 GT3 and SOSIP N276D) presenting a CD4bs epitope that is increasingly more native-like than the priming immunogen eOD-GT8. (B) BG505 core-GT3 and SOSIP-GT3 were also designed to minimize off-target responses. (C) Conservation of T-help between sequential immunogens. When using BG505 core-GT3 NP, the nanoparticle base is shared between the prime and first boost, while BG505 core gp120 is shared between the first and second boost. When using BG505 SOSIP-GT3, a PADRE peptide is conserved between the prime and first boost. (D) Affinity of germline-reverted (GLrev) and mature VRC01-class antibodies for BG505 core-GT3. See also Figures S1, S2, and S3 and Tables S1 and S2.

We sought to develop a boost immunogen to activate eOD-GT8 60-mer-induced memory B cells, cause the formation of new germinal centers, and select for a pool of more highly mutated memory B cells that could subsequently be boosted by native-like N276(−) trimers. To achieve this goal, we designed “bridging” molecules, which, while still germline-targeting, display a more native CD4bs epitope in order to drive maturation toward mature VRC01-class bnAbs. We allowed fewer overall mutations than in eOD-GT8 while retaining native VRC01-class contact residues wherever possible. Engineered mutations at non-contact positions were selected to improve affinity for germline-reverted (GLrev) Abs via conformational stabilization or removal of occluding glycans (Figure 1A). Two design platforms were chosen: core gp120 and SOSIP native-like trimer. The core gp120 platform was selected as an intermediate presentation of the CD4bs, in terms of epitope completeness and steric restriction, between the minimal eOD and the native-like trimer; use of core gp120 would also minimize boosting of off-target responses, as core gp120 shares little exposed, non-glycosylated surface with eOD or the native-like trimer beyond the CD4bs epitope (Figure 1B) (Jardine et al., 2013, Jardine et al., 2015, McGuire et al., 2014, McGuire et al., 2016). The SOSIP trimer platform was used to test the effect of including more native-like epitope and steric access restrictions in this bridging boost immunogen. The BG505 strain was selected for both core gp120 and SOSIP platforms, primarily for the purpose of conserving T-help with the subsequent boost of BG505 SOSIP N276D (Figure 1C), although changing to BG505 from the HXB2 strain used as the base strain for eOD-GT8 was also considered potentially advantageous for minimizing off-target responses (Figure 1B). To conserve T-help with the eOD-GT8 60-mer prime, we planned to display the core-gp120 on the same nanoparticle (lumazine synthase) as eOD-GT8 (Jardine et al., 2013, Jardine et al., 2015), thus using the underlying nanoparticle for conserved T-help. For the trimer platform, we added an exogenous T-help epitope (PADRE) (Alexander et al., 1994, Alexander et al., 1998) to the C terminus of both the eOD-GT8 60-mer prime and the SOSIP boost (Figure 1C).

**Figure S1:**
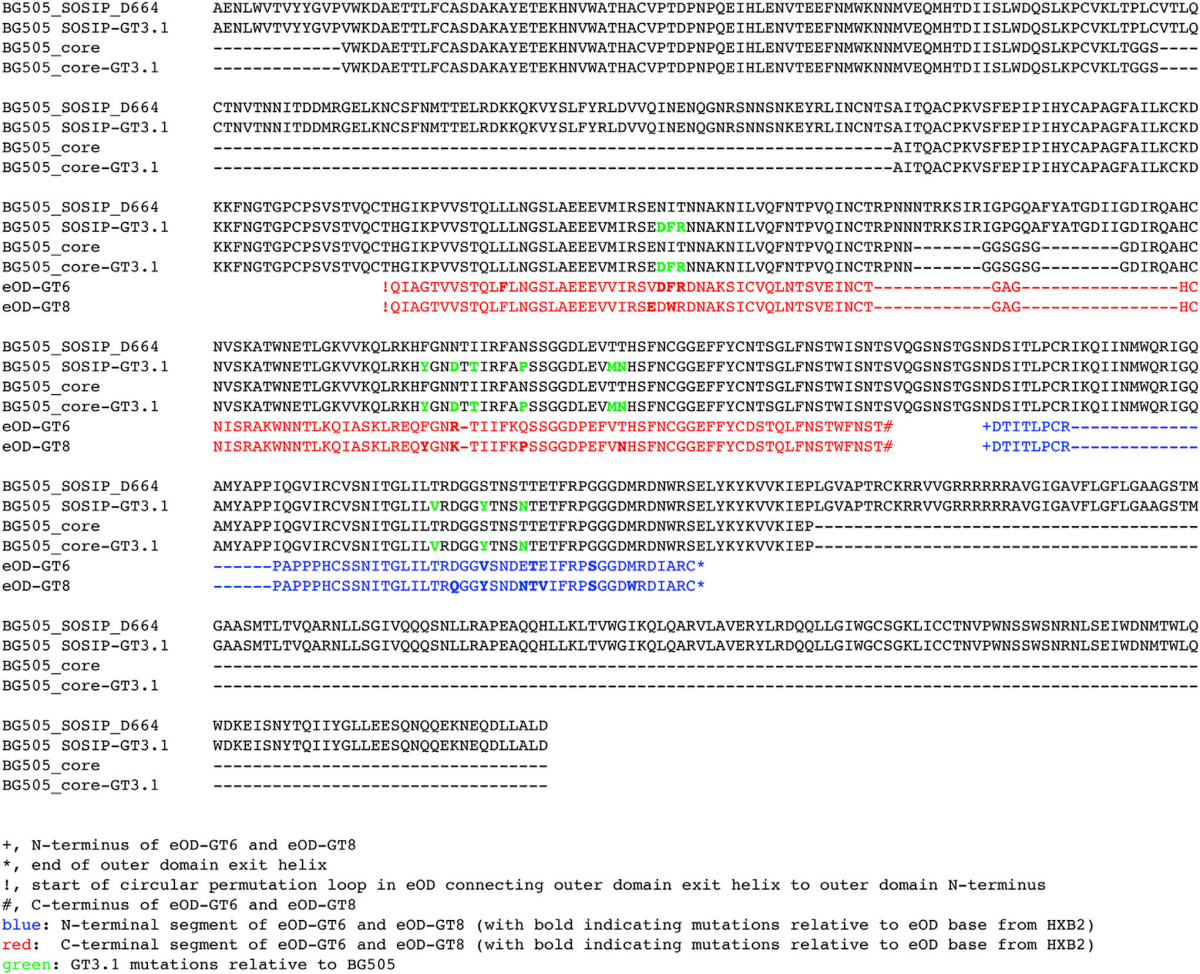
Development of Boosting Immunogen GT3.1, Related to Figure 1 Sequence alignment of BG505 SOSIP.664, BG505 GT3.1 SOSIP, BG505 core, BG505 GT3.1 core, GT6, and GT8. Engineered mutations in GT3.1 are highlighted in green.

**Figure S2:**
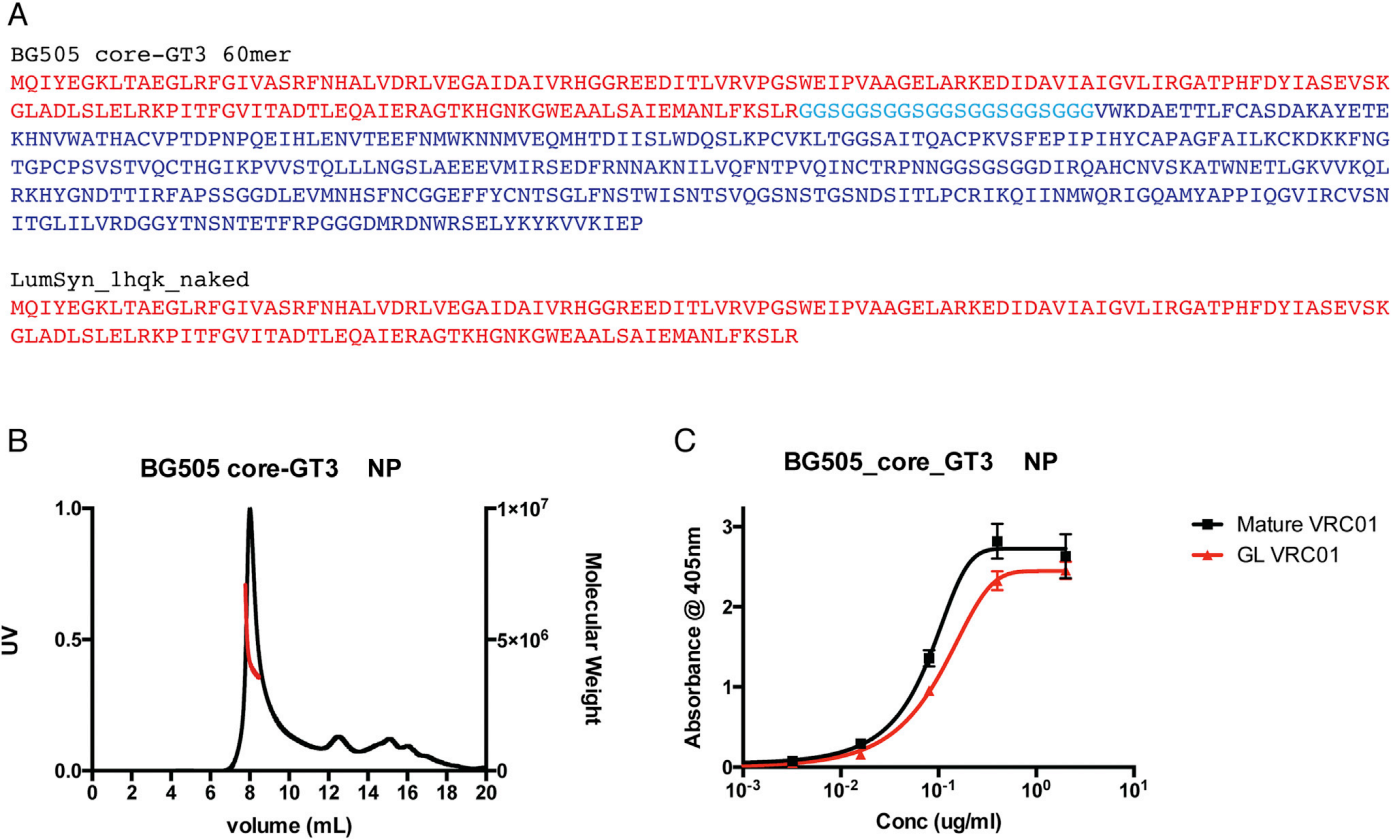
Sequence and Characterization of BG505 Core-GT3.1 Nanoparticles, Related to Figure 1 (A) Sequences of the two genes co-transfected to produce BG505 core-GT3 nanoparticles (NPs). Co-transfection is 80% BG505 core GT3 60mer and 20% LumSyn_1hqk_naked. In the BG505 core-GT3 60-mer gene, BG505 core-GT3 (blue) is fused to the lumazine synthase gene (red) via a flexible linker (cyan) as shown. (B) SECMALS analysis of BG505 core-GT3 NP. (C) ELISA binding of mature VRC01 (black) or GLRev VRC01 (red) to BG505 core-GT3 NP.

Protein engineering was initially carried out by yeast display directed evolution on the core gp120 platform. We generated combinatorial libraries of core BG505 gp120 containing mutations that we had previously noted in the development of core BaL-GT1 and eOD-GT8 (Jardine et al., 2013, Jardine et al., 2016a). These libraries were displayed on yeast and screened for binding to a panel of GLrev VRC01-class mAbs. Three rounds of optimization resulted in BG505 core-GT3 (Figure S1), a construct with modest affinity for GLrev VRC01-class Abs (Figure 1D). Unlike eOD-GT8, which displayed similar affinity for both GLrev and mature VRC01-class mAbs (Jardine et al., 2016a), BG505 core-GT3 bound VRC01-class bnAbs with >1,000-fold higher affinity than their GLrev counterparts. Thus, BG505 core-GT3 displayed a strong affinity gradient for mature bnAbs over GLrev Abs and was therefore promising as a boost to select productive somatic hypermutation (Figure 1D). We produced nanoparticles of BG505 core-GT3 by genetic fusion to lumazine synthase, as previously reported for eOD-GT6 and eOD-GT8 (Jardine et al., 2013, Jardine et al., 2015). However, to accommodate the larger core-gp120, nanoparticles included ∼20 mol% “naked” lumazine synthase. Thus, we estimate that there were ∼48 copies of core-GT3 displayed on the nanoparticles. BG505 core-GT3 nanoparticles (NPs) displayed approximately the expected molecular weight in solution, according to SECMALS analysis, and maintained antigenicity for GLrev VRC01-class Abs (Figure S2).

**Figure S3:**
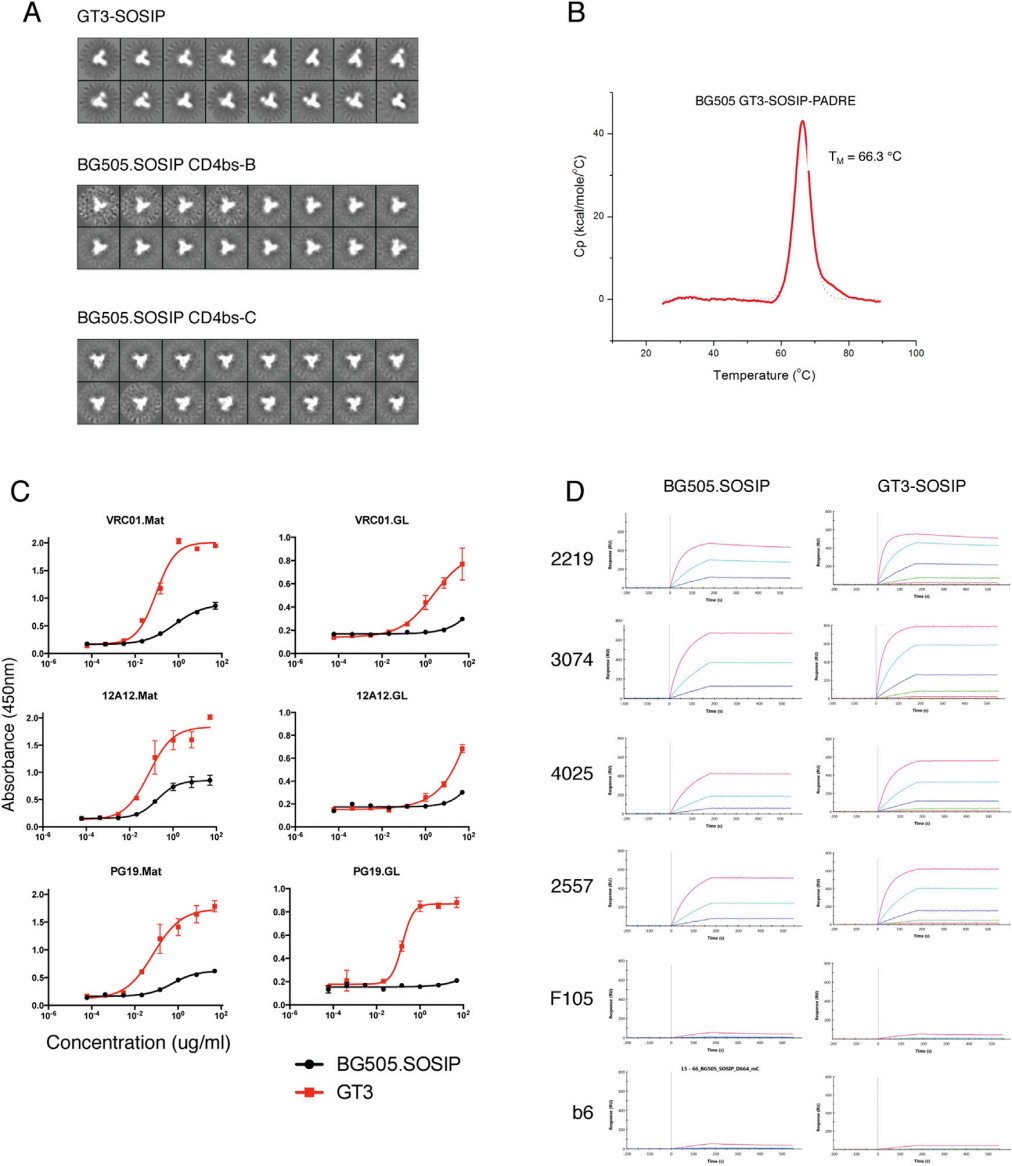
Characterization and Structural Analysis of BG505 GT3.1 SOSIP and ABC SOSIP Trimers, Related to Figure 1 (A) Negative-stain 2D class average images of the GT3.1 SOSIP, CD4bs-B SOSIP and CD4bs-C SOSIP are shown. 37% of GT3.1 SOSIP trimers were classified as closed native-like trimers, 63% as open native-like trimers and 0% as non-native-like trimers. (B) DSC thermogram of GT3.1 SOSIP. (C) ELISA analysis of mature and GLRev VRC01-class Ab binding to BG505 SOSIP and BG505 GT3.1 SOSIP. (D) SPR sensograms of BG505 SOSIP and BG505 GT3.1 SOSIP as analytes and non-nAbs IgGs as ligands. Maximum analyte concentrations were 990 nM and 1.3 uM for BG505 SOSIP and BG505 GT3 SOSIP, respectively, and lower concentrations teseted were 4-fold dilutions.

To generate a native-like trimer variant of GT3 with more native-like epitope features and CD4bs steric access restrictions, we transferred the BG505 core-GT3 mutations onto BG505.D664 SOSIP and added a C-terminal PADRE epitope, resulting in BG505 SOSIP-GT3-PADRE, from now on referred to as BG505 SOSIP-GT3 (Figure S1). Overall, BG505 SOSIP-GT3 was trimeric by SECMALS and showed an antigenic profile similar to BG505 SOSIP.D664, with the added ability to bind VRC01-class GLrev Abs and with a similar VRC01-class affinity gradient as for BG505 core-GT3 (Figure S3). The melting temperatures of BG505-SOSIP-GT3 (66.3°C) and BG505 SOSIP.D664 (66.7°C), were similar. By negative-stain EM analysis, BG505 SOSIP-GT3 was indistinguishable from BG505 SOSIP.D664 (Figure S3).

Thus, to develop a sequential immunization scheme with considerations of gradual epitope change toward a native configuration, T-help conservation, and minimizing the boosting of off-target responses, two boost candidates were designed to follow the eOD-GT8 60-mer and precede the BG505 SOSIP N276D native-like trimer.

### Prime and Boosting of VRC01-gH Mice

**Figure 2:**
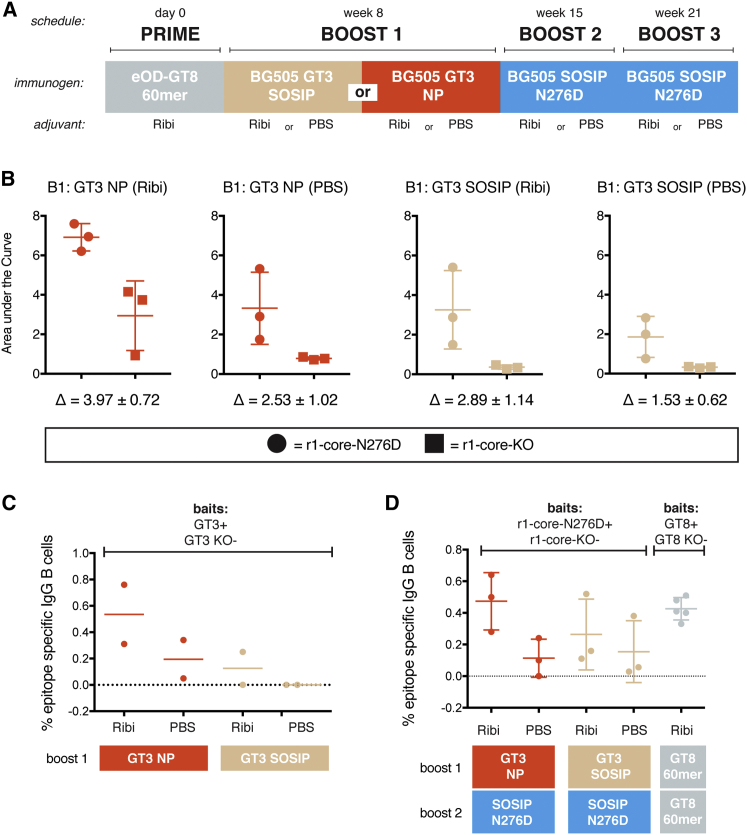
Immunization of VRC01 gH Mice (A) Schedule for priming and boosting VRC01 gH mice. Each immunization group consisted of five animals, two of which were sacrificed following the first boost (BG505 GT3 SOSIP or BG505 GT3 NP), with the remaining three animals receiving the entire immunization schedule before being sacrificed. (B) ELISA binding of VRC01 gH mouse serum following boost 3. The immunogen used for boost 1 (B1) and the adjuvant used for all three boosts is shown for each plot. Binding to r1-core-N276D (filled circles) and r1-core-KO (filled squares) is represented as area under the curve, and the difference ± SEM between binding to r1-core-N276D and r1-core-KO is shown beneath each plot. Each point represents a single animal that received the entire immunization schedule, of which there are three per immunization group. The value of each point is the mean of three technical replicates. The error bars represent mean ± SD. (C) Frequency of epitope-specific IgG memory B cells in VRC01-gH mice 14 days following boost 1, as measured by binding to GT3 and lack of binding to GT3-KO. Each point represents a single animal that was sacrificed following the first boost, of which there are two per group. The mean for each group is indicated by a single horizontal bar. (D) Frequency of epitope-specific IgG memory B cells after the full immunization regimen, measured by binding to r1-core-N276D and lack of binding to r1-core-KO (for animals boosted twice with GT3 and SOSIP N276D) or binding to GT8 and lack of binding to GT8-KO (for animals that were boosted twice with GT8). Each point represents a single animal that received the entire immunization schedule, of which there were three per group. The error bars represent mean ± SD. See also Figures S4 and S5.

To quantify the ability of BG505 core-GT3 NP and BG505 SOSIP-GT3 to recall VRC01-class precursor B cells primed with eOD-GT8 60-mer, we sequentially immunized the VRC01 gH mouse with eOD-GT8 60-mer, BG505 core-GT3 NP, and BG505 SOSIP N276D trimer according to the immunization schedule described in Figure 2A. Twenty VRC01 gH mice were primed with eOD-GT8 60-mer prime followed by either BG505 core-GT3 NP or BG505 SOSIP-GT3 boost. Eight mice were sacrificed following the initial boost, while 12 mice (six boosted with core-GT3 NP and six boosted with SOSIP-GT3) received two additional boosting immunizations of BG505 SOSIP N276D.

**Figure S4:**
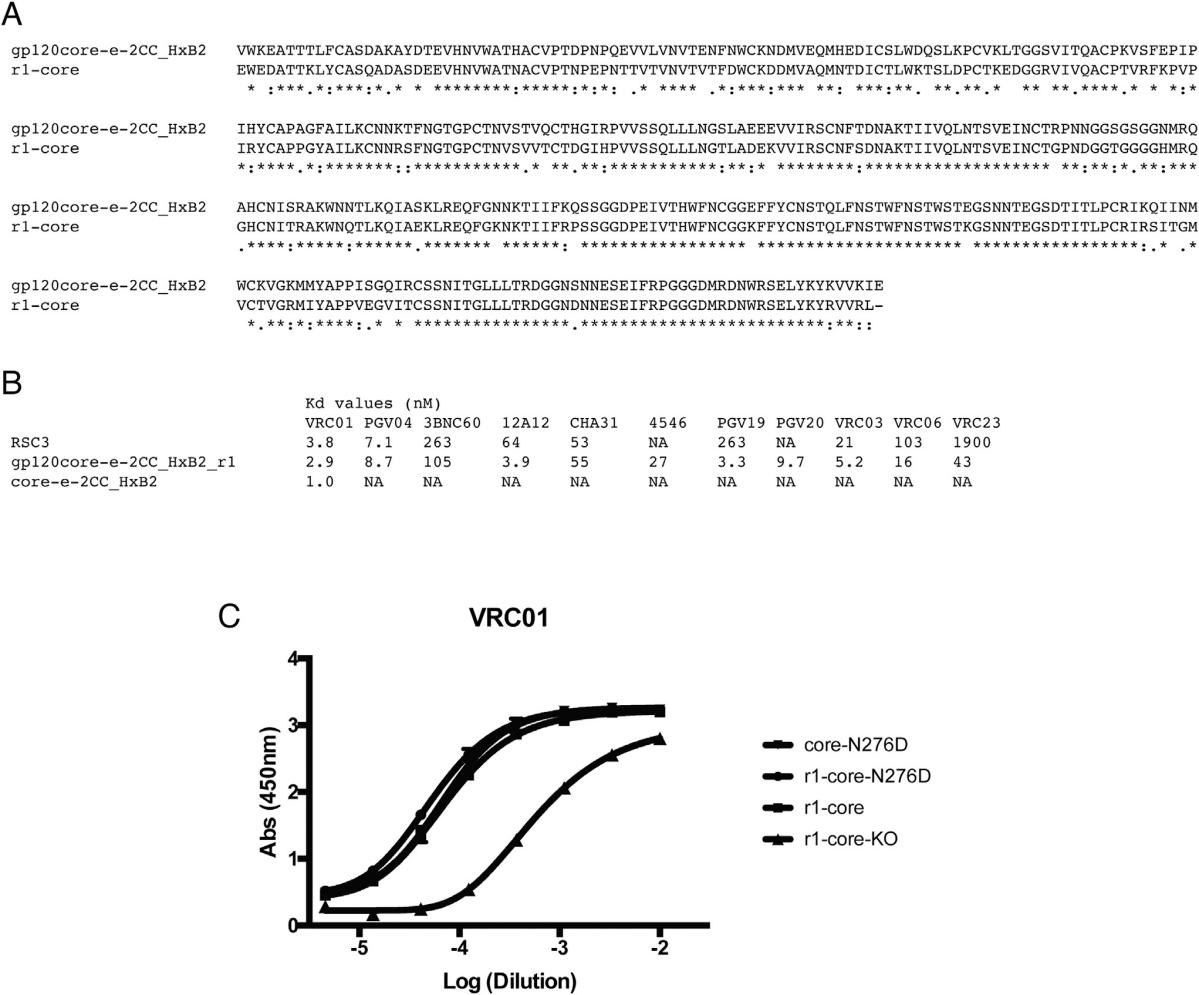
Sequence and Antigenicity of the r1-Core Resurfaced Core gp120, Related to Figure 2 (A) Sequence alignment of r1-core with gp120core-e-2CC HxB2. The r1-core is derived from the core-e-2CC HxB2 protein that we previously described (Jardine et al., 2015) and is also known as gp120core-e-2CC_HxB2_r1. A total of 78 surface positions have been modified, out of 358 total residues. Mutations are classified in the third row of the alignment, with “”: denoting a conservative mutation between amino acids with similar physicochemical properties; ” ” denoting a non-conservative mutation; and “.” denoting a semi-conservative mutation. (B) Dissociation constants for core-e-2CC HxB2, r1-core, and RSC3 (Wu et al., 2010) with VRC01-class bnAbs. (C) ELISA binding of mature VRC01 to core-N276D (core-e-2CC HxB2 with the N276D mutation; inverted triangles), r1-core-N276D (circles), r1-core (squares) and r1-core-KO (triangles). Starting concentration of mature VRC01 was 2 μg/ml.

For a serological probe, we developed a resurfaced HXB2 core gp120 (r1-core-N276D) with improved VRC01-class antigenicity compared to RSC3 (Wu et al., 2010) (Figure S4). The resurfacing should minimize the binding of antibodies induced by our immunization protocol against epitopes other than the VRC01-class epitope. A VRC01-class epitope knockout variant (r1-core-KO) with substantially depressed affinities for VRC01-class bnAbs was also engineered, by adding the mutations D368R and N279A in the CD4bs (Li et al., 2007, Li et al., 2011).

**Figure S5:**
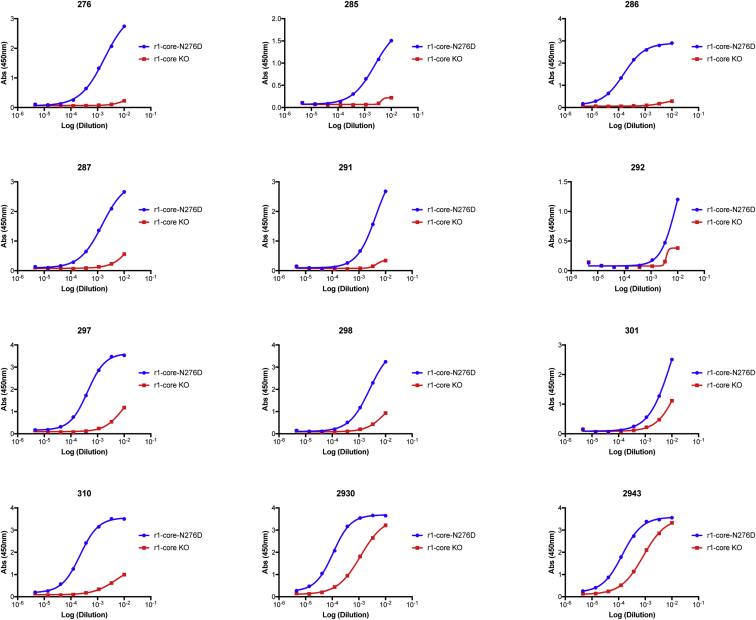
Serum Binding to r1-Core and r1-Core-KO, Related to Figure 2 Serum binding to r1-core (red, also referred to as HXcore) and r1-core-KO (blue, also referred to as HXcore KO). Representative binding curves from twelve mice are shown. Best fit curves for binding to r1-core and r1-core-KO were used to compute the AUC shown in Figure 2B.

Following the third boost, we evaluated serum antibody binding to r1-core-N276D and r1-core-KO (Figures 2B and S5). Areas under the curve (AUC) were calculated for each serum sample, and the differences in AUC between r1-core-N276D and r1-core-KO are shown in Figure 2B. Although there was substantial intragroup variation, the greatest differential was observed for BG505 core-GT3 NP delivered with Ribi adjuvant. Similar epitope-specific serum responses were seen in mice boosted with BG505 core-GT3 NP without adjuvant and BG505 SOSIP-GT3 delivered with Ribi adjuvant. In contrast, BG505 SOSIP-GT3 delivered without adjuvant produced relatively modest responses and the smallest difference in reactivity between r1-core-N276D and r1-core-KO.

These differences in serum antibody responses were mirrored in the frequencies of epitope-specific memory B cells. Splenocytes and lymph nodes from immunized animals were harvested and stained for IgG memory B cells. Single cells were then antigen-sorted by flow cytometry using the baits listed in Figures 2C and 2D. When comparing IgG memory B cell frequencies following the first boost, BG505 core-GT3 NP delivered in Ribi adjuvant showed the highest frequency of epitope-specific memory B cells (Figure 2C). The same group showed the highest frequency of epitope-specific memory B cells upon completion of the full boosting schedule (Figure 2D).

### Selection of Productive Mutations with Boosting

**Figure 3:**
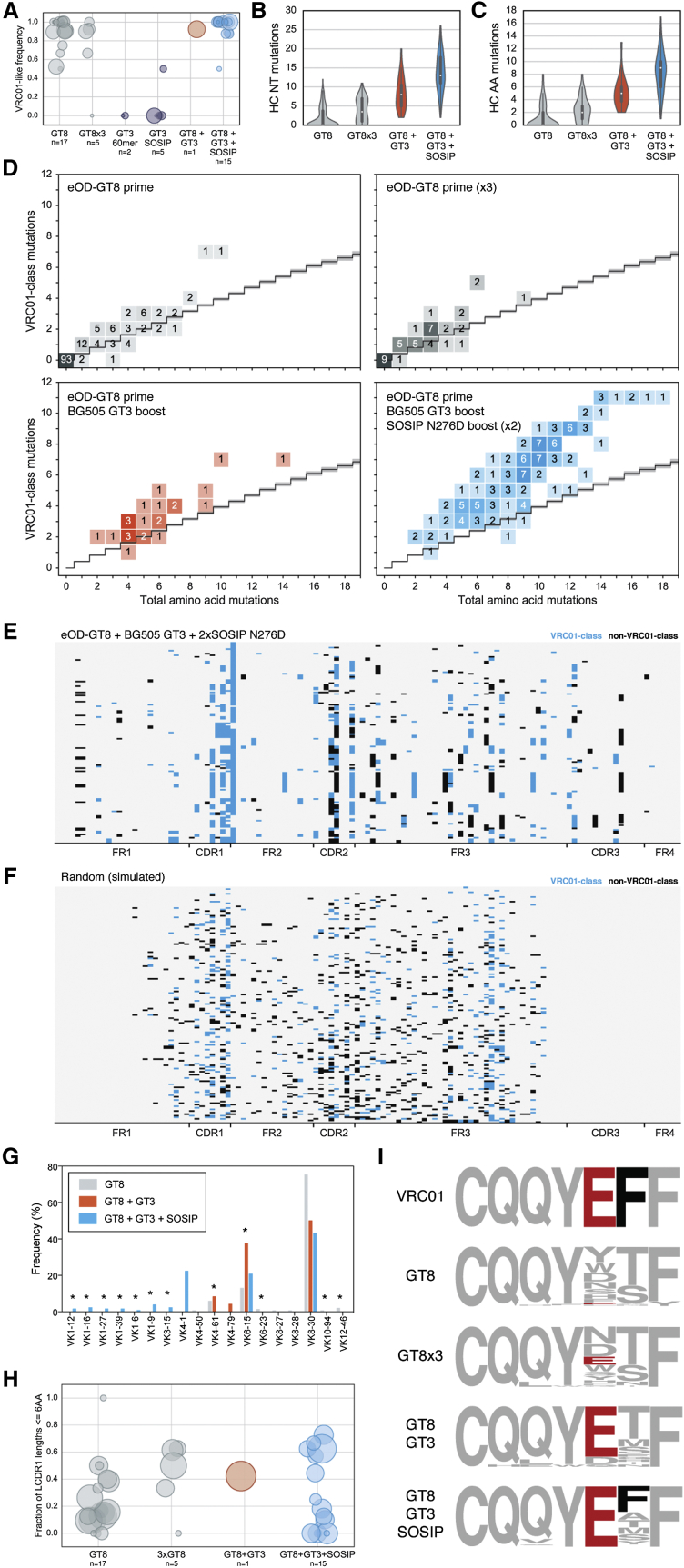
Genetic Maturation of Immunogen-Induced Antibodies (A) Frequency of VRC01-class (VH1-2 heavy chain and a 5AA long LCDR3) antibodies among all paired heavy-light sequences recovered per mouse, for different mice in each immunization group. The numbers below each immunization group indicate the number of mice from which paired sequences were recovered. Each bubble represents antibody sequences from a single mouse and the area of the bubble is proportional to the total number of mAb sequences recovered. (B) Frequency of heavy-chain nucleotide mutations for all sequences from all animals in each immunization group. (C) Same as (B) but for amino acid mutations. (D) Two-dimensional histograms of the number of VRC01-class-like amino mutations (defined as those shared with VRC01, PGV04, PGV20, VRC-CH31, 3BNC60, and 12A12) (Jardine et al., 2015) versus the total number of amino acid mutations, for VH genes in all VRC01-class pairs recovered from each immunization group. The frequency of VRC01-class mutations expected by random SHM (black line) is shown on each plot, as well as the 95% confidence interval (gray shading). (E) Locations of VRC01-class mutations (blue) and other mutations (black) within heavy chains of all 130 VRC01-class pairs recovered from 15 animals receiving the complete immunization schedule (eOD-GT8 60-mer, BG505 GT3, and SOSIP N276D 2x). Positions at which the antibody sequence was identical to GLRev VRC01 are colored light gray. (F) Locations of VRC01-class mutations (blue) and other mutations (black) from 150 sequences randomly selected from a pool of 2,000 artificial antibody sequences that represent random SHM activity layered on the VRC01 gH sequence. Synthetic mutations were only generated in the variable gene region; hence, the lack of synthetic mutations in CDR3 and FR4 (see STAR Methods for a more detailed explanation). (G) Frequency of mouse light chain variable genes from paired VRC01-class sequences recovered from different immunization groups. Light-chain V-genes with germline-encoded short (≤6AA) LCDR1s are indicated with asterisks. See also Table S3. (H) Frequency of light chains encoding a short (≤6AA) LCDR1 for each mouse in different immunization groups. The numbers below each group indicate the number of mice from which paired sequences were recovered. Each bubble represents a single animal, and the area of the bubble is proportional to the total number of VRC01-like pairs. (I) LCDR3 sequence logos from each paired VRC01-class mAb in each immunization group compared to LCDR3 of VRC01. Increasing convergence on critical LCDR3 residues found in VRC01 is highlighted.

To determine whether consecutive boosting with tailored immunogens selects for productive mutations, we divided the immunized VRC01 gH mice into two test groups: (1) mice that received only the eOD-GT8 60-mer prime and a single boost of BG505 GT3 (either SOSIP or NP), and (2) mice that received the complete immunization protocol outlined in Figure 2A (with either GT3 SOSIP or NP as the initial boost). We also analyzed four control groups of VRC01-gH mice that received the following regimens: (1) a single immunization of eOD-GT8 60-mer, (2) three successive immunizations of eOD-GT8 60-mer, (3) a single priming immunization of BG505 core-GT3 NP, and (4) a single priming immunization of BG505 SOSIP-GT3. Overall, we recovered 681 heavy-chain sequences, 753 light-chain sequences, and 430 paired heavy-light-chain sequences. In animals primed with eOD-GT8 60-mer, the majority of paired sequences were VRC01-like (defined as using VH1-2 and encoding a 5AA LCDR3) (Figure 3A). In contrast, only one of seven mice primed with BG505-GT3 (either SOSIP or NP) generated VRC01-like antibodies, demonstrating the necessity of a high-affinity germline-targeting prime (Figure 3A). Although single or multiple immunizations with eOD-GT8 60-mer alone failed to induce substantial SHM, heterologous boosting resulted in significantly mutated antibody sequences, with the most mutated heavy-chain sequence containing 17 amino acid mutations (17.3%) and a mean amino-acid mutation frequency of 8.6% in mice that were primed with eOD-GT8 60-mer and boosted with BG505 GT3 and twice with BG505 SOSIP N276A (Figures 3B and 3C).

We next examined whether vaccine-induced SHM was progressing toward mature VRC01. For each VH1-2 sequence, we determined the total number of amino-acid mutations and the number of amino-acid mutations shared with a panel of VRC01-class mAbs (VRC01, PGV04, PGV20, VRC-CH31, 3BNC60, and 12A12) (Jardine et al., 2015) (Figure 3D). In order to compare the observed frequency of shared VRC01-class mutations to the frequency expected by random SHM, we performed extremely deep antibody repertoire sequencing on two healthy HIV-naive individuals and used that information to compute the frequency of randomly incorporated VRC01-class mutations in human VH1-2 antibody sequences (Figure 3D). In animals given a single or triple immunization of eOD-GT8 60-mer alone, the frequency of VRC01-class mutations was similar to that expected by chance. This finding was anticipated, because eOD-GT8 has similar affinity for GLrev and mature VRC01-class antibodies (Jardine et al., 2016a) and likely places minimal selective pressure on the incorporation of VRC01-class mutations. In animals boosted with more native-like immunogens, however, VRC01-class mutations were selected much more frequently than would be expected by chance: 126 of 130 VRC01-like heavy/light-chain paired antibody sequences from animals boosted with GT3 and SOSIP N276A (2x) incorporated VRC01-class mutations at a frequency higher than the calculated 95% confidence interval of random SHM. The stepwise increase in SHM after each boost and the general failure of GT3 to prime VRC01-class responses suggested efficient recall of previously stimulated responses rather than recruitment of primarily naive B cells upon each boost.

We also interrogated vaccine-elicited light chains for evidence of maturation toward mature VRC01. This analysis is less straightforward than with heavy chains, because the light chains were derived from mouse germline genes that would not be expected to follow the same maturation pathway as human VRC01-class light chains. Instead, we assessed two critical features: the distribution of LCDR1 lengths and sequence convergence in LCDR3. VRC01-class bnAbs encode relatively short LCDR1 loops (2AA–8AA) by utilizing germline variable genes with short (6AA–8AA) LCDR1s and in some cases with further shortening by SHM-associated deletions (Table S3) (West et al., 2012, Zhou et al., 2013). Although indels are relatively rare (Briney et al., 2012a, Jardine et al., 2016b) and likely will be difficult to elicit consistently by vaccination (Jardine et al., 2016b), we were keenly interested in whether sequential immunization could select antibodies with short LCDR1s. Mice at all stages of the immunization program had VRC01-like antibodies encoding 6AA LCDR1s (Figure 3G), accomplished through the use of a variety of light-chain genes with germline-encoded short LCDR1s (Figure 3H). We also noted strong sequence convergence of immunogen-elicited antibodies on a critical glutamate residue (Glu96) found in LCDR3 of VRC01 and most other VRC01-class bnAbs (Figure 3I) (West et al., 2012, Zhou et al., 2015). While Glu96 was rarely present in light chains recovered from GT8-primed mice (3%), the frequency increased upon successive boosts, and every antibody recovered from mice primed with GT8 and boosted with GT3 and SOSIP N276D (2x) contained the critical LCDR3 glutamate. Therefore, mirroring the data obtained from heavy-chain sequences, sequential boosting successfully recalled primed VRC01-like precursors and selected for the incorporation of specific genetic features that evolved light chains toward VRC01-class bnAbs.

### Broad Serum Neutralization of N276A Viruses

**Figure 4:**
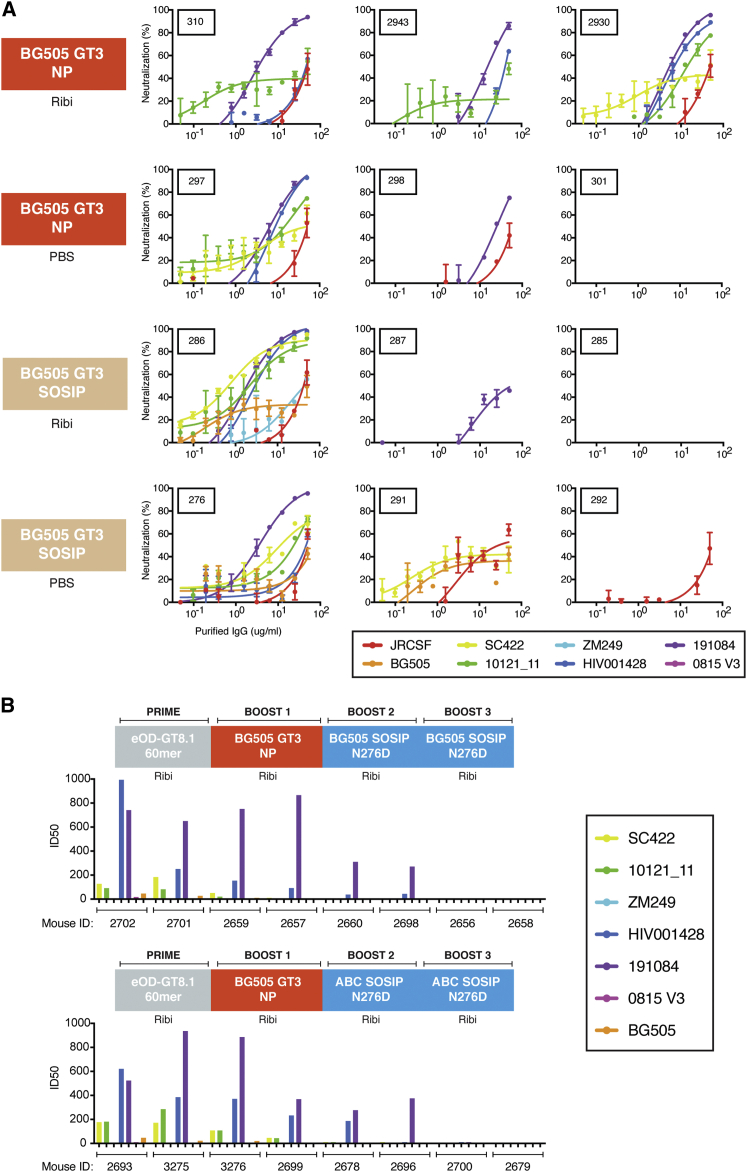
Serology of Immunized VRC01 gH Mice (A) Neutralization curves of total IgG purified from sera of immunized mice. Twelve mice were primed with eOD-GT8 60-mer and boosted with either core-GT3 NP or GT3 SOSIP (adjuvanted with either Ribi or PBS) followed by an additional two boosts with BG505 SOSIP N276D (with either Ribi or PBS). The leftmost column describes the immunization regimen of the three plots immediately to the right. Each neutralization plot shows neutralization activity against an 8-virus panel of near-native (N276A) virus isolates. Neutralization values with error bars are mean ± SD for two measurements. Total IgG concentrations are shown in μg/ml. Best fit curves were calculated with GraphPad Prism. (B) Sixteen additional VRC01 gH mice were primed with eOD-GT8 60-mer and boosted with BG505 core-GT3 NP, followed by two boosts with either BG505 SOSIP N276D in Ribi (top) or ABC SOSIP N276D cocktail in Ribi (bottom). Purified serum IgG was screened against a 7-virus panel of near-native (N276A) virus isolates, and ID_50_ values are plotted for each mouse, as reciprocal serum titers.

Following immunization, purified serum IgG from each of the 12 mice receiving the entire immunization schedule was screened on an 8-virus cross-clade indicator panel of near-native (N276A) HIV isolates (Figure 4A). Ten of the 12 mice demonstrated neutralization of at least one heterologous near-native isolate, and six of the mice developed cross-clade neutralization. Five of six mice boosted with BG505 core-GT3 60-mer developed cross-clade neutralization compared to three of six mice boosted with BG505 SOSIP-GT3. Interestingly, although all boosting immunogens were derived from BG505, only two mice acquired detectable neutralizing activity against BG505 N276D. Although five of six animals boosted with BG505 GT3 NP developed cross-clade neutralization compared to only two of six animals immunized with BG505 SOSIP-GT3, the two animals that exhibited the broadest neutralization were both boosted with BG505 SOSIP-GT3.

To verify the results of the first immunization experiment, we performed a repeat immunization with an additional 16 VRC01 gH mice, divided into two groups. All mice in the repeat experiment were primed with eOD-GT8 60-mer and boosted with BG505 core-GT3 NP according to the schedule in Figure 4B. One group of eight mice received BG505 SOSIP N276D for the final two boosts, while the other group received a cocktail of SOSIP N276D isolates, one each from clades A, B, and C (ABC SOSIP N276D; Figure S3; Table S2). We did not observe any difference between the two groups of mice with respect to serum neutralization, with six mice in each group developing cross-clade neutralization on a 7-virus panel of near-native isolates (identical to the 8-virus panel used in the first round of immunizations, but without JRCSF) (Figure 4B). Only three mice in each group developed neutralizing activity against BG505 N276A, suggesting that using a single isolate for the final two boosts did not focus the immune response on the immunizing isolate. This is also in agreement with the first immunizations, as three of the six BG505 N276A-boosted mice that displayed neutralizing activity developed heterologous neutralization without acquiring detectable autologous neutralizing activity.

### mAbs from Immunized Mice Broadly Neutralize Near-Native Viruses

**Figure 5:**
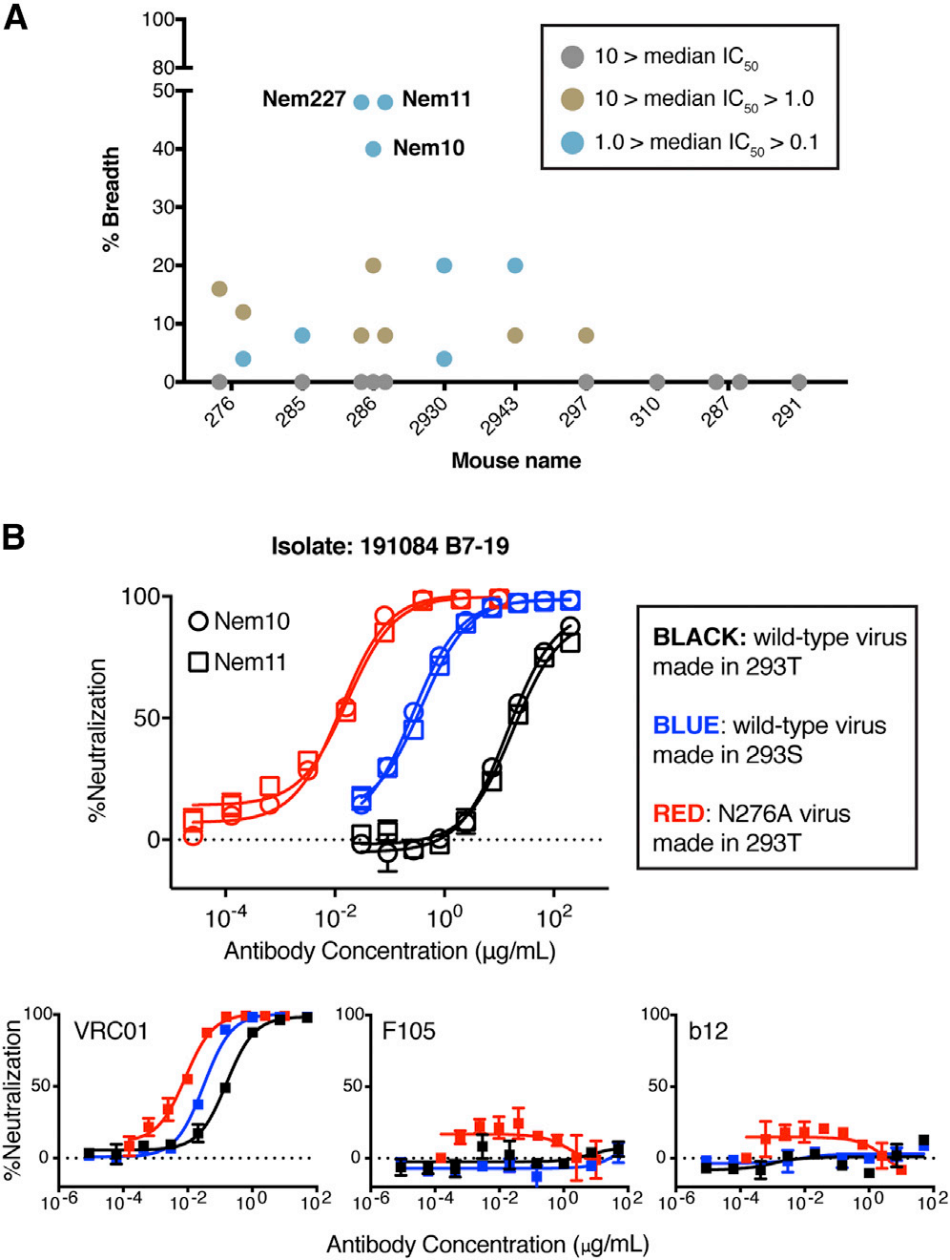
Neutralization by mAbs from Immunized Mice (A) Neutralization breadth and potency of mAbs isolated from several mice receiving the entire immunization program and screened on a 25-virus cross-clade panel of near-native (N276A) isolates. (B) Neutralization activity of two mAbs isolated from mouse 286. Nem10 (circles) and Nem11 (squares) neutralized wild-type 191084 B7-19 virus grown in 293T cells (black) or 293S cells (blue), as well as N276A virus grown in 293T cells (red), with potency highest against N276A virus. In the bottom three panels, neutralization curves for mature VRC01, F105, and b12 are shown for comparison. Neutralization values with error bars are mean ± SD for two measurements. Best fit curves were calculated with GraphPad Prism. See also Figure S6 and Table S4.

**Figure S6:**
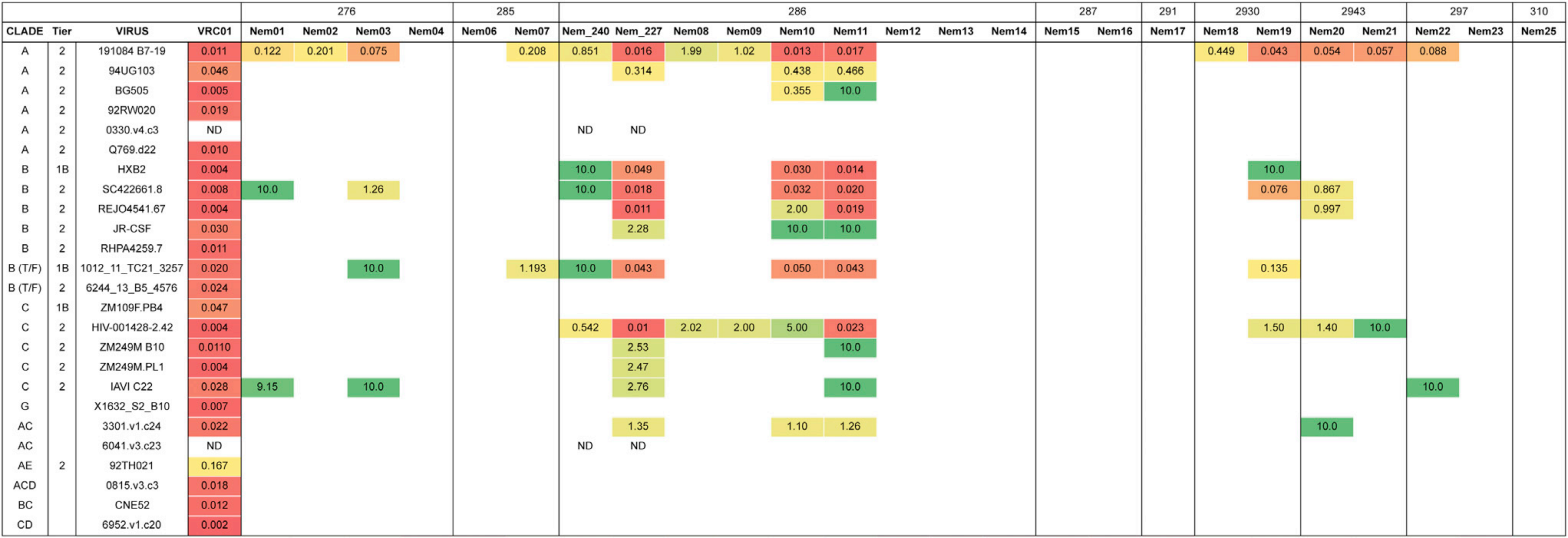
Broad Neutralization of Near-Native Viruses by VRC01-gH mAbs, Related to Figure 5 Neutralization of a 25-virus panel consisting of near-native (N276A) viruses, with IC50 values shown in μg/ml. Antibodies are grouped by the mouse from which they were isolated. Blank entries indicate lack of neutralization (> 10 μg/ml), and antibody/virus combinations that were not tested are noted (ND). Neutralization by mature VRC01 is shown for comparison. Tier phenotypes for the parent viruses lacking the N276A mutation were taken from (Binley et al., 2004, Seaman et al., 2010, Sellhorn et al., 2012, Hraber et al., 2014).

We expressed 25 mAbs from eight VRC01 gH mice that were primed with GT8 and boosted with GT3 and SOSIP and tested them for neutralizing activity on the 7-virus N276A virus panel described earlier. Generally, neutralization breadth of mAbs from each mouse correlated with the breadth of serum neutralization, although one mouse (285) that showed no detectable serum neutralization produced a single mAb with moderate neutralization activity. Antibodies from several mice showed broad neutralization on the 7-virus near-native panel, and mAbs from mouse 286 neutralized select isolates with potency comparable to mature VRC01 (Figure S6). The mAbs were then screened on a larger 25-virus panel of N276A isolates (Figure 5A). The broadest antibodies all came from a single mouse that also had the broadest serum neutralization (286, first boosted by SOSIP-GT3). Three mAbs from this mouse (Nem227, Nem10, and Nem11) were surprisingly broad and potent, neutralizing up to 48% of the viruses on the 25-virus panel with a median IC_50_ of <1 μg/ml.

Because Nem10 and Nem11 neutralized 191084 B7-19 N276A with potencies comparable to mature VRC01, we tested the ability of these two mAbs to neutralize the fully native version of 191084 B7-19, a tier 2 virus (Sellhorn et al., 2012, Hraber et al., 2014). Both antibodies were able to neutralize wild-type virus grown in 293S cells, albeit with lower potency (Figure 5B), suggesting that the mAbs can accommodate the N276 glycan and other CD4bs glycans when they are of reduced size. Critically, both antibodies also showed weak neutralizing activity against fully native virus grown in 293T cells (Figure 5B), which in comparison to the high potency against 293T-grown N276A virus suggests that the antibodies are capable of accommodating the N276 glycan to a degree but at some energy cost to binding that precludes high-affinity interaction. No neutralizing activity was detected for these mAbs against the other native viruses in the 25-member panel grown in 293T cells. It is important to note that enhanced potency against virus isolates lacking the N276 glycan site is consistent with a VRC01-like response. Mature VRC01 is substantially more potent against isolates lacking the N276 glycan, but removal of the N276 glycan site does not make tier 2 viruses more susceptible to the non-VRC01-class bnAb b12, to CD4 IgG2, or to the CD4-binding site-directed non-neutralizing antibodies b6 or F105 (Jardine et al., 2016b). In summary, an immunization program consisting of an eOD-GT8 60-mer prime followed by boosts with BG505 GT3 (NP or SOSIP) and SOSIP N276D elicited antibodies with broad neutralization on a panel of near-native, tier-2 virus isolates, moderate neutralization of one wild-type virus grown in 293S cells and weak neutralization of one fully native tier-2 virus.

## Discussion

VRC01-class bnAbs are prototypical examples of the neutralizing anti-HIV response that an optimal vaccine would elicit: they are broadly and potently neutralizing; multiple VRC01-class bnAbs have been shown to be protective against infection in animal models; and VRC01-class naive B cell precursors are likely to be present at a reasonable frequency in a large fraction of the population thus offering targets to initiate vaccine elicitation. However, induction of such responses remains a massive challenge in part because VRC01-class bnAbs display exceptionally high levels of somatic mutation and GLrev versions of these antibodies have no detectable affinity for all native-like HIV Env molecules tested thus far. A multi-step reductionist vaccine strategy has the potential to address both of these issues: an engineered germline-targeting prime can activate VRC01-class precursors and generate boostable VRC01-class memory B cells, and successive heterologous boosts with increasingly native-like immunogens can produce additive rounds of somatic mutation and gradually refine the ability of maturing antibodies to recognize native HIV Env. Development of minimally mutated variants of VRC01-class antibodies that retain broad and potent neutralizing activity has further raised expectation that a VRC01-like antibody response is achievable by vaccination (Georgiev et al., 2014, Jardine et al., 2016b).

We have previously reported a germline-targeting immunogen, eOD-GT8 60-mer, capable of activating germline precursors of VRC01-class bnAbs. Because eOD-GT8 60-mer requires a highly engineered CD4bs epitope to activate VRC01-class precursors, antibodies elicited by priming with eOD-GT8 60-mer do not show any detectable affinity for native HIV Env (Jardine et al., 2015). Therefore, the lack of intermediate immunogens to bridge the gap between the engineered CD4bs in eOD-GT8 60-mer and the native CD4bs in native-like trimers like BG505 SOSIP has remained an obstacle to the elicitation of neutralizing VRC01-class antibody responses. Here, we report the development of core-GT3 and SOSIP-GT3, vaccine components designed to shepherd primed VRC01-class precursors toward intermediate VRC01-class function. Boosting VRC01-gH mice with BG505-GT3 (NP or SOSIP) and then with BG505 SOSIP N276D resulted in the elicitation of highly mutated antibodies with a significant fraction of the mutations shared with mature VRC01-class bnAbs. Enrichment of VRC01-class mutations in heavy chains following immunization, and convergence of light-chain CDR3 residues toward the sequence of mature VRC01, indicate that boosting with BG505-GT3 and SOSIP N276D establishes strong selective pressure on specific VRC01-class mutations and places these antibodies on a maturation trajectory consistent with partially mature VRC01-class antibodies.

Although boosted VRC01 gH mice showed broad neutralization on a panel of N276A viruses, neutralization of fully native virus containing the N276 glycan site was limited to a single heterologous tier 2 isolate and was substantially less potent. While the weak neutralization of fully native HIV indicates that there is still significant work to be done before we are able to elicit a truly functional broadly neutralizing response, these data strongly suggest that the elicited responses are VRC01-class antibodies of intermediate maturity. Mature VRC01, in contrast to non-neutralizing mAbs that target the CD4bs, neutralizes N276A viruses much more potently than fully native viruses, so the limited activity of the elicited mAbs against fully native viruses containing the N276 glycan site may simply be a normal feature of partially mature VRC01-class antibodies (Jardine et al., 2016b, Kong et al., 2016). Indeed, the observed preference for N276A is not unexpected, as neither the prime nor any of the boosting immunogens contain the N276 glycan site. In mature VRC01-class bnAbs, the N276 glycan is accommodated by use of a short LCDR1 loop, either germline-encoded or generated through SHM-mediated LCDR1 deletions (Jardine et al., 2016b, West et al., 2012, Zhou et al., 2013). Encouragingly, we observed a significant fraction of elicited mAbs with LCDR1 lengths matching those of germline-encoded short LCDR1s in mature VRC01-class bnAbs.

The relatively low VRC01-class precursor frequency and substantial competition from other clones in the VRC01 gH mouse pose a relatively high bar for elicitation of VRC01-class responses. Thus, the ability to recall VRC01-class precursors and drive maturation toward mature VRC01-class function validates the reductionist sequential immunization strategy and represents a significant milestone in HIV vaccine development.

## STAR★Methods

### Key Resources Table

**Table:** 

REAGENT or RESOURCE	SOURCE	IDENTIFIER
**Antibodies**		

anti-GL7 PerCP-eFluor710	eBioSciences	Cat#:46-5902-82
anti-CD38 PE-Cy7	eBioSciences	Cat#:25-0381-82
anti-CD19 BUV396	Beckton Dickinson	Cat#:563557
anti-B220 BUV396	Beckton Dickinson	Cat#:563793
anti-CD8 APC-Cy7	Beckton Dickinson	Cat#:560182
anti-CD4 APC-Cy7	Beckton Dickinson	Cat#:560181
anti-F4/80 APC-Cy7	BioLegend	Cat#:123118
anti-Gr-1 APC-Cy7	Beckton Dickinson	Cat#:557661
anti-CD11c APC-Cy7	Beckton Dickinson	Cat#:561241
anti-IgM BV786	Beckton Dickinson	Cat#:564028
anti-IgD BV605	Beckton Dickinson	Cat#:563003

**Chemicals, Peptides, and Recombinant Proteins**		

eOD-GT8 60mer	Jardine et al., 2015, Jardine et al., 2016a	N/A
BG505 core-GT3 nanoparticles	This paper	N/A
BG505 SOSIP-GT3	This paper	N/A
BG505 SOSIP N276D	This paper	N/A
eOD-GT8	Jardine et al., 2015, Jardine et al., 2016a	N/A
eOD-GT8 KO	Jardine et al., 2015, Jardine et al., 2016a	N/A
r1-core-N276D	This paper	N/A
r1-core-KO	This paper	N/A
ABC SOSIP N276D cocktail	This paper	N/A

**Deposited Data**		

Antibody sequence data and analysis scripts	This paper	https://github.com/briney/VRC01gH-GT3; GenBank: KX779470–KX779519 and KX808478–KX808481

**Experimental Models: Cell Lines**		

TZM-bl cells	NIH AIDS reagent program	Cat#:8129
HEK293S GnTI- cells	ATCC	Cat#:CRL-3022

**Experimental Models: Organisms/Strains**		

VRC01 gH mouse	Jardine et al., 2015	N/A

**Sequence-Based Reagents**		

Murine PCR primers	Tiller et al., 2008; Table S4	N/A
Human VH1-2 PCR primer	Sok et al., 2014; Table S4	N/A

**Software and Algorithms**		

AbStar		https://www.github.com/briney/abstar
Clonify	Briney et al., 2016	https://www.github.com/briney/clonify
Sickle		https://www.github.com/najoshi/sickle
Cutadapt	Martin, 2011	https://github.com/marcelm/cutadapt
PANDAseq	Masella et al., 2012	https://github.com/neufeld/pandaseq

### Contact for Reagent and Resource Sharing

Further information and requests for reagents may be directed to, and will be fulfilled by the corresponding author William R. Schief (schief@scripps.edu).

### Experimental Model and Subject Details

#### Mice

All animal studies were approved by The Scripps Research Institute Institutional Animal Care and Use Committee. VRC01 gH transgenic mice have been described previously (Jardine et al., 2015). Mice were 6-8 weeks old at the time of the first immunization. 63% of the mice were male and 37% were female, with each immunization group containing a mix of male and female mice. Mice were housed in a specific pathogen free environment.

#### Healthy, HIV-Negative Human PBMCs

Leukopaks from two heathy, HIV-negative individuals (a 28 year-old female and a 33 year-old male) were obtained from a commercial vendor (AllCells) using a protocol approved by the Institutional Review Boards of both AllCells and The Scripps Research Institute. Peripheral blood mononuclear cells (PBMCs) were isolated using gradient centrifugation.

### Method Details

#### Protein Production and Purification

eOD-GT8 and BG505 GT3 monomers and NPs were produced and purified as described previously (Jardine et al., 2015). BG505 SOSIP D664 and BG505-GT3 SOSIP gp140 trimers were produced in mammalian cells (HEK293F) by co-transfection of the trimer gene and furin protease, at a trimer to furin ratio of 2:1. The pre-transfected cells were maintained in 293 Freestyle media (Life Technologies) in a humidified 37°C C02 incubator (8%), rotating at 135rpm at a density of ∼2.4 × 10^6^ cells/ml. The genes were transfected using 293fectin (Invitrogen) and harvested 4-5 days later. The cells were centrifuged at 4000rpm for 15min, filtered using 0.2 μm filter (Millipore) and a protease inhibitor was added at ratio of 1ml per liter of supernatant (Protease Arrest, GBiosciences). The supernatants were purified by nickel affinity purification using His-Trap columns (GE), starting with a wash buffer (20mM Imidizole, 500 mM NaCl, 20 mM Na2HPO4) and mixing with elution buffer (500 mM Imidizole, 500 mM NaCl, 20 mM Na2HPO4) using a linear gradient. The trimers were then purified by semi-analytical size exclusion chromatography on a S200Increase 10-300 column (GE) in HBS (10mM HEPES, 150mM NaCl). The trimer fractions were pooled, concentrated to 1mg/ml by using Ultracel 30K centrifugal spin concentrators (Millipore) and measuring concentration on a NanoDrop 2000c Spectrophotometer using the absorption signal at 280 nm, frozen in thin-walled PCR tubes using liquid nitrogen, and then stored at −80°C. BG505 SOSIP trimers produced by this in-house process have been thawed and analyzed by SECMALS, SPR, differential scanning calorimetry, and electron microscopy and have been found to possess the native-like antigenic profile, thermal stability and closed trimeric structure that have been reported by others for BG505 SOSIP purified by an antibody-affinity column followed by SEC (Julien et al., 2013, Lyumkis et al., 2013, Pancera et al., 2014, Sanders et al., 2013).

The thermostable self-assembling lumazine sythase 60-mer (PDB ID: 1HQK), previously described for displaying eOD-GT6 (Jardine et al., 2013) and eOD-GT8 (Jardine et al., 2015), was adapted to display stabilized extended HIV gp120 core (gp120core-e) antigens from different strains. Initial expression tests with gp120core-e fused to the 1hqk sequence (gp120core-e-1hqk) via various length linkers failed to produce fully assembled particles despite high expression levels of the subunits. We then tested co-transfection with a plasmid encoding only the base subunit of lumazine synthase to insert spacers into the 60-mer thereby reducing the crowding on the surface. Of all gp120core-e-1hqk/base 1hqk plasmid DNA combinations tested (95/5, 90/10, 85/15, 80/20, 66/33, 50/50, 33/60), an 80% gp120core-e-1hqk and 20% base 1hqk mixture produced the highest proportion of assembled 60mers.

#### Antibody Production

Antibodies were expressed in the pFUSEss human IgG1 vector (Invitrogen). Heavy- and light-chain plasmids were cotransfected (1:1 ratio) in 293 FreeStyle cells using 293fectin (Invitrogen). Transfections were performed according to the manufacturer’s protocol, and antibody supernatants were harvested 4-5 days after transfection. Antibody supernatants were purified over Protein A Sepharose 4 Fast Flow (GE healthcare) columns, eluted with 0.1M citric acid (pH 3.0), and dialyzed against phosphate-buffered saline.

#### Surface Plasmon Resonance

Kinetics and affinities of antibody-antigen interactions were measured as described previously (Jardine et al., 2016a). Briefly, we measured kinetics and affinities of antibody-antigen interactions on a ProteOn XPR36 (Bio-Rad) using GLC Sensor Chip (Bio-Rad) and 1x HBS-EP+ pH 7.4 running buffer (20x stock from Teknova, Cat. No H8022) supplemented with BSA at 1mg/ml. We followed the Human Antibody Capture Kit instructions (Cat. No BR-1008-39 from GE) to prepare chip surfaces for ligand capture. In a typical experiment, about 6000 RU of capture antibody was amine-coupled in all 6 flow cells of the GLC Chip. Regeneration was accomplished using 3M Magnesium Chloride with 180 s contact time and injected four times per each cycle. Raw sensograms were analyzed using ProteOn Manager software (Bio-Rad), including interspot and column double referencing, and either Equilibrium fits or Kinetic fits with Langmuir model, or both, were employed when applicable. Analyte concentrations were measured on a NanoDrop 2000c Spectrophotometer using Absorption signal at 280 nm.

#### Single-Cell Sorting by Flow Cytometry

Mice spleen and lymph node samples were processed for single B cell sorting based on previously described methods (Sok et al., 2014, Tiller et al., 2008, Wu et al., 2011), with slight modifications. Mouse spleens were stained with primary fluorophore-conjugated antibodies to murine CD4, CD8, F4/80, CD11c, Gr-1, CD19, B220, IgD, IgM, CD38, and GL7 markers. Memory B cells were selected for the phenotype CD19+, B220+, CD4-, CD8-, F4/80-, CD11c-, Gr-1-, IgM-, IgD-, while CD38 and GL7 markers were monitored to measure germinal center B cell frequencies (CD38-, GL7+). For antigen-specific staining, 50 nM of biotinylated AviTag r1-core-N276D monomer and its CD4bs KO variant (r1-core-KO) were coupled to Streptavidin-AF488 and Streptavidin-PE (Life Technologies) in equimolar ratios, respectively. B cells of interest were single-cell sorted into 96 well plates containing lysis buffer on a BD FACSAria Fusion sorter and immediately stored at −80°C (Sok et al., 2014, Tiller et al., 2008, Wu et al., 2011).

#### Single B Cell RT-PCR, Gene Amplification, and Cloning

Reverse transcription and subsequent PCR amplification of heavy and light chain variable genes were performed using SuperScript III (Life Technologies) according to published protocols (Sok et al., 2014, Tiller et al., 2008, Wu et al., 2011). All PCR reactions were performed in 25 μl volume with 2.5 μl of cDNA transcript using HotStar Taq DNA polymerase master mix (QIAGEN) and mixtures of previously described primers (Tiller et al., 2008) that were supplemented with a human VH1-2 primer (Jardine et al., 2015). Second round nested-PCR reactions were performed using Phusion proof reading polymerase (NEB). Two additional rounds of PCR were performed using primers with barcodes specific to the plate number and well location as well as adapters appropriate for sequencing on an Illumina MiSeq. This reaction was performed in a 25 μl volume with HotStar Taq DNA polymerase master mix (QIAGEN). Amplified IgG heavy- and light-chain variable regions were sequenced on an Illumina MiSeq (600-base v3 reagent kit; Illumina) and reads corresponding to the same plate/well location were combined into consensus sequences. Germline assignment and sequence annotation of the consensus sequences was performed with AbStar (https://.github.com/briney/abstar).

#### ELISA Assays

Ninety-six-well ELISA plates were coated overnight at 4°C with 50 uL PBS containing 100 ng of antigen per well. The wells were washed four times with PBS containing 0.05% Tween 20 and blocked with 3% BSA at room temperature for 1 hr. Serial dilutions of sera were then added to the wells, and the plates were incubated at room temperature for 1 hr. After washing four times, goat anti-mouse IgG F(ab’_)2_ conjugated to alkaline phosphatase (Pierce), diluted 1:1000 in PBS containing 1% BSA and 0.025% Tween 20, was added to the wells. The plate was incubated at room temperature for 1 hr, washed four times, and the plate was developed by adding 50 uL of alkaline phosphatase substrate (Sigma) to 5 ml alkaline phosphatase staining buffer (pH 9.8), according to the manufacturer’s instructions. The optical density at 405 nm was read on a microplate reader (Molecular Devices). ELISA protocol for mAbs was as follows. ELISA plates were coated overnight at 4°C with 25 μl of 4 μg/ml anti-His Ab (Epitope TagAntibody His.H8, MA1-21315). The wells were washed 5 times with PBST (PBS with 0.2% Tween 20) and blocked with 5% milk at RT for 1 hr. The wells were washed 5 times with PBST. mAb were diluted to 10 μg/mL in 0.1% milk PBST then added to the plates and incubated at RT for 1 hr. After washing 5 times in PBST, goat anti-human HRP (Jackson) was diluted 1:5000 in 0.1% milk PBST, then 25 μl was added to each well and the plate was incubated at RT for 1hr. After washing 5 times in PBST, the plate was developed by adding 50 μl TMB ELISA solution (Thermofisher) and then 50 μl sulfuric acid stop solution after 10 min. The optical density at 450 nm was read on a microplate reader (Molecular Devices).

#### Envelope Mutations

Mutations were introduced by site-directed mutagenesis using the QuikChange site-directed mutagenesis kit (Stratagene) and mutants were verified by Sanger DNA sequencing.

#### Pseudovirus Production and Neutralization Assays

To produce pseudoviruses, plasmids encoding Env were co-transfected with an Env-deficient genomic backbone plasmid (pSG3ΔEnv) in a 1:2 ratio with the transfection reagent Fugene 6 (Promega). Pseudoviruses were harvested 72 hr post transfection for use in neutralization assays. Neutralizing activity was assessed using a single round of replication pseudovirus assay and TZM-bl target cells, as described previously (Li et al., 2005, Walker et al., 2011). Briefly, TZM-bl cells were seeded in a 96-well flat bottom plate at a concentration of 20,000 cells/well. The serially diluted virus/antibody mixture, which was pre-incubated for 1 hr, was then added to the cells and luminescence was quantified 48 hr following infection via lysis and addition of Bright-GloTM Luciferase substrate (Promega). To determine IC_50_ values, serial dilutions of mAbs were incubated with virus and the dose-response curves were fitted using nonlinear regression.

#### Antibody NGS on HIV-Negative Donors

Leukopaks were obtained from two healthy, HIV-negative individuals (AllCells) and peripheral blood mononuclear cells (PBMCs) were isolated by gradient centrifugation. PBMCs from each donor were separated into aliquots of 500 m cells and total RNA was extracted separately from each PBMC aliquot (RNeasy Maxi Kit, QIAGEN). In quadruplicate, 10uL of each RNA aliquot was separately amplified in 100uL RT-PCR reactions (OneStep RT-PCR Kit, QIAGEN) using previously reported primers (Briney et al., 2012b) and with the following cycling conditions: 55°C for 30 min; 94°C for 5 min; 25 cycles of 94°C for 30 s, 55°C for 30 s, 72°C for 2 min; 72°C for 7 min. RT-PCR reactions were purified using 0.8 volumes of SPRIselect magnetic beads (Beckman-Coulter Genomics) and replicate RT-PCR reactions were eluted together in 50ul of water. In duplicate reactions for each pooled RT-PCR sample, Illumina sequencing adapters and sample-specific indexes were added during a second round of PCR using 2uL of purified RT-PCR product in 100uL of total reaction volume (HotStarTaq Plus; QIAGEN) and using the following thermal cycling program: 94°C for 5 min; 10 cycles of 94°C for 30 s, 55°C for 30 s, 72C for 2 min; 72°C for 7 min. Indexed PCR products were purified using 75uL of SPRIselect beads and eluted in 50uL of water. Samples from each donor were quantified using fluorometry (Qubit; Life Technologies), pooled at approximately equimolar concentrations and each sample pool was requantified. The end result was two pools of samples, each pool corresponding to a single subject and consisting of 18-20 separately barcoded samples that represent the amplification product of approximately 500 million PBMCs. Sequencing was then performed on an Illumina HiSeq (HiSeq Rapid SBS Kit v2, 500 cycles).

#### Processing of NGS Sequence Data

Using the AbStar analysis pipeline (https://github.com/briney/abstar), raw sequencing reads were quality trimmed with Sickle (https://github.com/najoshi/sickle), adapters were removed with cutadapt (Martin, 2011), and paired reads were merged with PANDAseq (Masella et al., 2012). Germline gene assignment and sequence annotation was performed with AbStar and output was deposited into a MongoDB database. For each sample, which represents the antibody sequences derived from approximately 500 m PBMCs, a non-redundant database of amino-acid sequences was created, including only heavy-chain sequences encoded by IGHV1-2. Because each PBMC aliquot was processed separately, redundant copies across samples represents independent occurrences of the same sequence and these redundancies were retained.

#### Synthetic Generation of Randomly Mutated VH1-2 Heavy-Chain Sequences

Separately for each subject, each IGHV1-2 heavy chain sequence was aligned to the AbStar-assigned germline allele of IGHV1-2 and the position and mutated residue of each mutation were noted. These mutations were then used to generate synthetically mutated antibody sequences based on the conditional probability of actually occurring somatic mutations. For example, if the first synthetic mutation was an Alanine at position 24 (24A), the probability distribution for the subsequent synthetic mutation was computed using NGS sequences that contain a naturally occurring 24A mutation. If the second mutation was 36F, then the probability distribution for the third synthetic mutation would be computed from NGS sequences with both 24A and 36F. Of note, prior mutations were excluded from the conditional probability distribution. This ensures that, for example, the 24A mutation will not happen a second time in the same sequence. It is also important to note that, due to technical limitations on sequencing length and the annealing location of amplification primers midway through the framework 1 region (FR1), mutations in the first portion of FR1 were not sampled and thus were not used in mutation probability calculations. This is evident in the lack of mutations near the start of synthetically generated antibody sequences (Figure 3F). Because most VRC01-class mutations occur in CDR1 and CDR2, it is not likely that excluding FR1 mutations had a significant effect on the overall frequency of randomly occurring VRC01-class mutations.

#### Design of CD4bs Native-like Trimer Cocktail

A five member CD4bs cocktail was engineered on gp120-core by analyzing the sequence diversity of HIV strains at VRC01-class epitope positions, which includes the V5 loop. Each member of the cocktail incorporates mutations from a single strain, and these five strains were chosen to best mimic the diversity of HIV at VRC01-class epitope positions. We next created a native-like trimer cocktail (ABC) by transferring the mutations from the gp120-core cocktail and adding new mutations found proximal to the PGV04 VRC01-class bnAbs in the Env trimer structure (PDB ID: 3J5M) as well as inclusion of the V2 loop. Three of the five trimers formed native-like structures and antigenic profiles and were used as boosting immunogens in the VRC01-gH mice.

#### Negative-Stain Electron Microscopy

BG505-based SOSIP trimers were analyzed by negative stain EM by adapting a previously published protocol (de Taeye et al., 2016).

#### Differential Scanning Calorimetry

MicroCal VP-Capillary differential scanning calorimeter (Malvern Instruments) was used for DSC measurements. The protein samples were diluted into HEPES buffer to a final concentration of 0.25 mg/ml. The experiment scanned from 20°C to 90°C at a scan rate of 90°C/h. Data were analyzed by buffer correction, normalization, and baseline subtraction (Origin 7.0).

### Quantification and Statistical Analysis

When computing the frequency of random incorporation of VRC01-class mutations, we iterated temporally through mutations (taking the first mutation from each sequence, then the first two mutations, etc) and determined the frequency of mutations from each synthetic antibody sequence that were VRC01-class. Using the range of VRC01-class frequencies at each step, we computed the mean frequency (shown as a black line Figure 3D) and the 95% confidence intervals (shown as gray shading surrounding the mean line in Figure 3D). Both the mean and 95% CI were computed in Python using the Numpy and Scipy packages. All other statistical calculations were performed in Graphpad Prism. The number of replicates, the type of data represented in figure plots, and a description of the statistical method are provided in the applicable figure legends.

### Data and Software Availability

#### Data Resources

The sequences of elicited antibodies reported in this paper have been deposited at GenBank: KX779470–KX779519 and KX808478–KX808481 as well as in the GitHub repository: https://github.com/briney/VRC01gH-GT3.

## Author Contributions

B.B., D.S., J.G.J., D.W.K., P.S., D.N., D.R.B., and W.R.S. planned and designed the experiments. B.B., D.S., J.G.J., D.W.K., P.S., S.M., R.J., O.K., N.d.V., F.S., K.M.L., A.R., M.J., K.L.S.-F., T.R.B., S.S., E.G., X.H., G.O., Y.A., M.K., and A.S. performed the experiments and analyzed data. B.B., D.S., J.G.J., D.W.K., D.R.B., and W.R.S. wrote the manuscript. All authors contributed to manuscript revisions. I.A.W., A.B.W., D.N., D.R.B., and W.R.S. supervised the research.

## References

[bib1] Alexander J., Sidney J., Southwood S., Ruppert J., Oseroff C., Maewal A., Snoke K., Serra H.M., Kubo R.T., Sette A. (1994). Development of high potency universal DR-restricted helper epitopes by modification of high affinity DR-blocking peptides. Immunity.

[bib2] Alexander J., Fikes J., Hoffman S., Franke E., Sacci J., Appella E., Chisari F.V., Guidotti L.G., Chesnut R.W., Livingston B., Sette A. (1998). The optimization of helper T lymphocyte (HTL) function in vaccine development. Immunol. Res..

[bib3] Andrabi R., Voss J.E., Liang C.-H., Briney B., McCoy L.E., Wu C.-Y., Wong C.-H., Poignard P., Burton D.R. (2015). Identification of common features in prototype broadly neutralizing antibodies to HIV envelope V2 apex to facilitate vaccine design. Immunity.

[bib4] Barouch D.H., Whitney J.B., Moldt B., Klein F., Oliveira T.Y., Liu J., Stephenson K.E., Chang H.-W., Shekhar K., Gupta S. (2013). Therapeutic efficacy of potent neutralizing HIV-1-specific monoclonal antibodies in SHIV-infected rhesus monkeys. Nature.

[bib5] Bhiman J.N., Anthony C., Doria-Rose N.A., Karimanzira O., Schramm C.A., Khoza T., Kitchin D., Botha G., Gorman J., Garrett N.J. (2015). Viral variants that initiate and drive maturation of V1V2-directed HIV-1 broadly neutralizing antibodies. Nat. Med..

[bib6] Binley J.M., Wrin T., Korber B., Zwick M.B., Wang M., Chappey C., Stiegler G., Kunert R., Zolla-Pazner S., Katinger H. (2004). Comprehensive cross-clade neutralization analysis of a panel of anti-human immunodeficiency virus type 1 monoclonal antibodies. J. Virol..

[bib69] Briney B., Le K., Zhu J., Burton D.R. (2016). Clonify: unseeded antibody lineage assignment from next-generation sequencing data. Sci. Rep..

[bib7] Briney B.S., Willis J.R., Crowe J.E. (2012). Location and length distribution of somatic hypermutation-associated DNA insertions and deletions reveals regions of antibody structural plasticity. Genes Immun..

[bib8] Briney B.S., Willis J.R., McKinney B.A., Crowe J.E. (2012). High-throughput antibody sequencing reveals genetic evidence of global regulation of the naïve and memory repertoires that extends across individuals. Genes Immun..

[bib9] Burton D.R., Hangartner L. (2016). Broadly neutralizing antibodies to HIV and their role in vaccine design. Annu. Rev. Immunol..

[bib10] Burton D.R., Stanfield R.L., Wilson I.A. (2005). Antibody vs. HIV in a clash of evolutionary titans. Proc. Natl. Acad. Sci. USA.

[bib11] Caskey M., Klein F., Lorenzi J.C.C., Seaman M.S., West A.P., Buckley N., Kremer G., Nogueira L., Braunschweig M., Scheid J.F. (2015). Viraemia suppressed in HIV-1-infected humans by broadly neutralizing antibody 3BNC117. Nature.

[bib12] de Taeye S.W., Moore J.P., Sanders R.W. (2016). HIV-1 envelope trimer design and immunization strategies to induce broadly neutralizing antibodies. Trends Immunol..

[bib13] Dimitrov D.S. (2010). Therapeutic antibodies, vaccines and antibodyomes. MAbs.

[bib14] Diskin R., Scheid J.F., Marcovecchio P.M., West A.P., Klein F., Gao H., Gnanapragasam P.N.P., Abadir A., Seaman M.S., Nussenzweig M.C., Bjorkman P.J. (2011). Increasing the potency and breadth of an HIV antibody by using structure-based rational design. Science.

[bib15] Diskin R., Klein F., Horwitz J.A., Halper-Stromberg A., Sather D.N., Marcovecchio P.M., Lee T., West A.P., Gao H., Seaman M.S. (2013). Restricting HIV-1 pathways for escape using rationally designed anti-HIV-1 antibodies. J. Exp. Med..

[bib16] Doria-Rose N.A., Schramm C.A., Gorman J., Moore P.L., Bhiman J.N., DeKosky B.J., Ernandes M.J., Georgiev I.S., Kim H.J., Pancera M., NISC Comparative Sequencing Program (2014). Developmental pathway for potent V1V2-directed HIV-neutralizing antibodies. Nature.

[bib17] Falkowska E., Le K.M., Ramos A., Doores K.J., Lee J.H., Blattner C., Ramirez A., Derking R., van Gils M.J., Liang C.-H. (2014). Broadly neutralizing HIV antibodies define a glycan-dependent epitope on the prefusion conformation of gp41 on cleaved envelope trimers. Immunity.

[bib18] Georgiev I.S., Rudicell R.S., Saunders K.O., Shi W., Kirys T., McKee K., O’Dell S., Chuang G.-Y., Yang Z.-Y., Ofek G. (2014). Antibodies VRC01 and 10E8 neutralize HIV-1 with high breadth and potency even with Ig-framework regions substantially reverted to germline. J. Immunol..

[bib19] Gorman J., Soto C., Yang M.M., Davenport T.M., Guttman M., Bailer R.T., Chambers M., Chuang G.-Y., DeKosky B.J., Doria-Rose N.A., NISC Comparative Sequencing Program (2016). Structures of HIV-1 Env V1V2 with broadly neutralizing antibodies reveal commonalities that enable vaccine design. Nat. Struct. Mol. Biol..

[bib20] Haynes B.F., Kelsoe G., Harrison S.C., Kepler T.B. (2012). B-cell-lineage immunogen design in vaccine development with HIV-1 as a case study. Nat. Biotechnol..

[bib21] Hessell A.J., Malherbe D.C., Pissani F., McBurney S., Krebs S.J., Gomes M., Pandey S., Sutton W.F., Burwitz B.J., Gray M. (2016). Achieving potent autologous neutralizing antibody responses against tier 2 HIV-1 viruses by strategic selection of envelope immunogens. J. Immunol..

[bib22] Hoot S., McGuire A.T., Cohen K.W., Strong R.K., Hangartner L., Klein F., Diskin R., Scheid J.F., Sather D.N., Burton D.R., Stamatatos L. (2013). Recombinant HIV envelope proteins fail to engage germline versions of anti-CD4bs bNAbs. PLoS Pathog..

[bib23] Hraber P., Korber B.T., Lapedes A.S., Bailer R.T., Seaman M.S., Gao H., Greene K.M., McCutchan F., Williamson C., Kim J.H. (2014). Impact of clade, geography, and age of the epidemic on HIV-1 neutralization by antibodies. J. Virol..

[bib24] Jardine J., Julien J.-P., Menis S., Ota T., Kalyuzhniy O., McGuire A., Sok D., Huang P.-S., MacPherson S., Jones M. (2013). Rational HIV immunogen design to target specific germline B cell receptors. Science.

[bib25] Jardine J.G., Ota T., Sok D., Pauthner M., Kulp D.W., Kalyuzhniy O., Skog P.D., Thinnes T.C., Bhullar D., Briney B. (2015). HIV-1 VACCINES. Priming a broadly neutralizing antibody response to HIV-1 using a germline-targeting immunogen. Science.

[bib26] Jardine J.G., Kulp D.W., Havenar-Daughton C., Sarkar A., Briney B., Sok D., Sesterhenn F., Ereño-Orbea J., Kalyuzhniy O., Deresa I. (2016). HIV-1 broadly neutralizing antibody precursor B cells revealed by germline-targeting immunogen. Science.

[bib27] Jardine J.G., Sok D., Julien J.-P., Briney B., Sarkar A., Liang C.-H., Scherer E.A., Henry Dunand C.J., Adachi Y., Diwanji D. (2016). Minimally mutated HIV-1 broadly neutralizing antibodies to guide reductionist vaccine design. PLoS Pathog..

[bib28] Julien J.-P., Cupo A., Sok D., Stanfield R.L., Lyumkis D., Deller M.C., Klasse P.J., Burton D.R., Sanders R.W., Moore J.P. (2013). Crystal structure of a soluble cleaved HIV-1 envelope trimer. Science.

[bib29] Kepler T.B., Liao H.-X., Alam S.M., Bhaskarabhatla R., Zhang R., Yandava C., Stewart S., Anasti K., Kelsoe G., Parks R. (2014). Immunoglobulin gene insertions and deletions in the affinity maturation of HIV-1 broadly reactive neutralizing antibodies. Cell Host Microbe.

[bib30] Kong L., Ju B., Chen Y., He L., Ren L., Liu J., Hong K., Su B., Wang Z., Ozorowski G. (2016). Key gp120 glycans pose roadblocks to the rapid development of VRC01-class antibodies in an HIV-1-infected Chinese donor. Immunity.

[bib31] Li M., Gao F., Mascola J.R.J., Stamatatos L., Polonis V.R.V., Koutsoukos M., Voss G., Goepfert P., Gilbert P., Greene K.M.K. (2005). Human immunodeficiency virus type 1 env clones from acute and early subtype B infections for standardized assessments of vaccine-elicited neutralizing antibodies. J. Virol..

[bib32] Li Y., Migueles S.A.S., Welcher B., Svehla K., Phogat A., Louder M.K.M., Wu X., Shaw G.M.G., Connors M., Wyatt R.T., Mascola J.R. (2007). Broad HIV-1 neutralization mediated by CD4-binding site antibodies. Nat. Med..

[bib33] Li Y., O’Dell S., Walker L.M., Wu X., Guenaga J., Feng Y., Schmidt S.D., McKee K., Louder M.K., Ledgerwood J.E. (2011). Mechanism of neutralization by the broadly neutralizing HIV-1 monoclonal antibody VRC01. J. Virol..

[bib34] Liao H.-X., Lynch R., Zhou T., Gao F., Alam S.M., Boyd S.D., Fire A.Z., Roskin K.M., Schramm C.A., Zhang Z., NISC Comparative Sequencing Program (2013). Co-evolution of a broadly neutralizing HIV-1 antibody and founder virus. Nature.

[bib35] Lyumkis D., Julien J.-P., de Val N., Cupo A., Potter C.S., Klasse P.J., Burton D.R., Sanders R.W., Moore J.P., Carragher B. (2013). Cryo-EM structure of a fully glycosylated soluble cleaved HIV-1 envelope trimer. Science.

[bib36] Ma B.-J., Alam S.M., Go E.P., Lu X., Desaire H., Tomaras G.D., Bowman C., Sutherland L.L., Scearce R.M., Santra S. (2011). Envelope deglycosylation enhances antigenicity of HIV-1 gp41 epitopes for both broad neutralizing antibodies and their unmutated ancestor antibodies. PLoS Pathog..

[bib37] Martin M. (2011). Cutadapt removes adapter sequences from high-throughput sequencing reads. EMBnet.

[bib38] Mascola J.R., Haynes B.F. (2013). HIV-1 neutralizing antibodies: understanding nature’s pathways. Immunol. Rev..

[bib39] Masella A.P., Bartram A.K., Truszkowski J.M., Brown D.G., Neufeld J.D. (2012). PANDAseq: paired-end assembler for illumina sequences. BMC Bioinformatics.

[bib40] McGuire A.T., Hoot S., Dreyer A.M., Lippy A., Stuart A., Cohen K.W., Jardine J., Menis S., Scheid J.F., West A.P. (2013). Engineering HIV envelope protein to activate germline B cell receptors of broadly neutralizing anti-CD4 binding site antibodies. J. Exp. Med..

[bib41] McGuire A.T., Dreyer A.M., Carbonetti S., Lippy A., Glenn J., Scheid J.F., Mouquet H., Stamatatos L. (2014). HIV antibodies. Antigen modification regulates competition of broad and narrow neutralizing HIV antibodies. Science.

[bib42] McGuire A.T., Gray M.D., Dosenovic P., Gitlin A.D., Freund N.T., Petersen J., Correnti C., Johnsen W., Kegel R., Stuart A.B. (2016). Specifically modified Env immunogens activate B-cell precursors of broadly neutralizing HIV-1 antibodies in transgenic mice. Nat Commun..

[bib43] Pancera M., McLellan J.S., Wu X., Zhu J., Changela A., Schmidt S.D., Yang Y., Zhou T., Phogat S., Mascola J.R., Kwong P.D. (2010). Crystal structure of PG16 and chimeric dissection with somatically related PG9: structure-function analysis of two quaternary-specific antibodies that effectively neutralize HIV-1. J. Virol..

[bib44] Pancera M., Zhou T., Druz A., Georgiev I.S., Soto C., Gorman J., Huang J., Acharya P., Chuang G.-Y., Ofek G. (2014). Structure and immune recognition of trimeric pre-fusion HIV-1 Env. Nature.

[bib45] Pantaleo G., Koup R.A. (2004). Correlates of immune protection in HIV-1 infection: what we know, what we don’t know, what we should know. Nat. Med..

[bib46] Pegu A., Yang Z.-Y., Boyington J.C., Wu L., Ko S.-Y., Schmidt S.D., McKee K., Kong W.-P., Shi W., Chen X. (2014). Neutralizing antibodies to HIV-1 envelope protect more effectively in vivo than those to the CD4 receptor. Sci. Transl. Med..

[bib47] Richman D.D., Wrin T., Little S.J., Petropoulos C.J. (2003). Rapid evolution of the neutralizing antibody response to HIV type 1 infection. Proc. Natl. Acad. Sci. USA.

[bib48] Rudicell R.S., Kwon Y.D., Ko S.-Y., Pegu A., Louder M.K., Georgiev I.S., Wu X., Zhu J., Boyington J.C., Chen X., NISC Comparative Sequencing Program (2014). Enhanced potency of a broadly neutralizing HIV-1 antibody in vitro improves protection against lentiviral infection in vivo. J. Virol..

[bib49] Sanders R.W., Derking R., Cupo A., Julien J.-P., Yasmeen A., de Val N., Kim H.J., Blattner C., de la Peña A.T., Korzun J. (2013). A next-generation cleaved, soluble HIV-1 Env trimer, BG505 SOSIP.664 gp140, expresses multiple epitopes for broadly neutralizing but not non-neutralizing antibodies. PLoS Pathog..

[bib50] Sanders R.W., van Gils M.J., Derking R., Sok D., Ketas T.J., Burger J.A., Ozorowski G., Cupo A., Simonich C., Goo L. (2015). HIV-1 VACCINES. HIV-1 neutralizing antibodies induced by native-like envelope trimers. Science.

[bib51] Scharf L., Scheid J.F., Lee J.H., West A.P., Chen C., Gao H., Gnanapragasam P.N.P., Mares R., Seaman M.S., Ward A.B. (2014). Antibody 8ANC195 reveals a site of broad vulnerability on the HIV-1 envelope spike. Cell Rep..

[bib52] Scheid J.F., Mouquet H., Ueberheide B., Diskin R., Klein F., Oliveira T.Y.K., Pietzsch J., Fenyo D., Abadir A., Velinzon K. (2011). Sequence and structural convergence of broad and potent HIV antibodies that mimic CD4 binding. Science.

[bib53] Seaman M.S., Janes H., Hawkins N., Grandpre L.E., Devoy C., Giri A., Coffey R.T., Harris L., Wood B., Daniels M.G. (2010). Tiered categorization of a diverse panel of HIV-1 Env pseudoviruses for assessment of neutralizing antibodies. J. Virol..

[bib54] Sellhorn G., Kraft Z., Caldwell Z., Ellingson K., Mineart C., Seaman M.S., Montefiori D.C., Lagerquist E., Stamatatos L. (2012). Engineering, expression, purification, and characterization of stable clade A/B recombinant soluble heterotrimeric gp140 proteins. J. Virol..

[bib55] Shingai M., Nishimura Y., Klein F., Mouquet H., Donau O.K., Plishka R., Buckler-White A., Seaman M., Piatak M., Lifson J.D. (2013). Antibody-mediated immunotherapy of macaques chronically infected with SHIV suppresses viraemia. Nature.

[bib56] Sok D., van Gils M.J., Pauthner M., Julien J.-P., Saye-Francisco K.L., Hsueh J., Briney B., Lee J.H., Le K.M., Lee P.S. (2014). Recombinant HIV envelope trimer selects for quaternary-dependent antibodies targeting the trimer apex. Proc. Natl. Acad. Sci. USA.

[bib57] Tiller T., Meffre E., Yurasov S., Tsuiji M., Nussenzweig M.C., Wardemann H. (2008). Efficient generation of monoclonal antibodies from single human B cells by single cell RT-PCR and expression vector cloning. J. Immunol. Methods.

[bib58] Walker L.M., Phogat S.K., Chan-Hui P.-Y., Wagner D., Phung P., Goss J.L., Wrin T., Simek M.D., Fling S., Mitcham J.L., Protocol G Principal Investigators (2009). Broad and potent neutralizing antibodies from an African donor reveal a new HIV-1 vaccine target. Science.

[bib59] Walker L.M., Huber M., Doores K.J., Falkowska E., Pejchal R., Julien J.-P., Wang S.-K., Ramos A., Chan-Hui P.-Y., Moyle M., Protocol G Principal Investigators (2011). Broad neutralization coverage of HIV by multiple highly potent antibodies. Nature.

[bib60] Wei X., Decker J.M., Wang S., Hui H., Kappes J.C., Wu X., Salazar-Gonzalez J.F., Salazar M.G., Kilby J.M., Saag M.S. (2003). Antibody neutralization and escape by HIV-1. Nature.

[bib61] West A.P., Diskin R., Nussenzweig M.C., Bjorkman P.J. (2012). Structural basis for germ-line gene usage of a potent class of antibodies targeting the CD4-binding site of HIV-1 gp120. Proc. Natl. Acad. Sci. USA.

[bib62] Wu X., Yang Z.-Y., Li Y., Hogerkorp C.-M., Schief W.R., Seaman M.S., Zhou T., Schmidt S.D., Wu L., Xu L. (2010). Rational design of envelope identifies broadly neutralizing human monoclonal antibodies to HIV-1. Science.

[bib63] Wu X., Zhou T., Zhu J., Zhang B., Georgiev I., Wang C., Chen X., Longo N.S., Louder M., McKee K., NISC Comparative Sequencing Program (2011). Focused evolution of HIV-1 neutralizing antibodies revealed by structures and deep sequencing. Science.

[bib64] Xiao X., Chen W., Feng Y., Zhu Z., Prabakaran P., Wang Y., Zhang M.-Y., Longo N.S., Dimitrov D.S. (2009). Germline-like predecessors of broadly neutralizing antibodies lack measurable binding to HIV-1 envelope glycoproteins: implications for evasion of immune responses and design of vaccine immunogens. Biochem. Biophys. Res. Commun..

[bib65] Zhou T., Georgiev I., Wu X., Yang Z.-Y., Dai K., Finzi A., Kwon Y.D., Scheid J.F., Shi W., Xu L. (2010). Structural basis for broad and potent neutralization of HIV-1 by antibody VRC01. Science.

[bib66] Zhou T., Zhu J., Wu X., Moquin S., Zhang B., Acharya P., Georgiev I.S., Altae-Tran H.R., Chuang G.-Y., Joyce M.G., NISC Comparative Sequencing Program (2013). Multidonor analysis reveals structural elements, genetic determinants, and maturation pathway for HIV-1 neutralization by VRC01-class antibodies. Immunity.

[bib67] Zhou T., Lynch R.M., Chen L., Acharya P., Wu X., Doria-Rose N.A., Joyce M.G., Lingwood D., Soto C., Bailer R.T., NISC Comparative Sequencing Program (2015). Structural repertoire of HIV-1-neutralizing antibodies targeting the CD4 supersite in 14 donors. Cell.

