# Supplementary material for: Tailored Immunogens Direct Affinity Maturation toward HIV Neutralizing Antibodies
Source: Cell. 2016 Sep 8;166(6):1459–1470.e11. doi: 10.1016/j.cell.2016.08.005 (PMC5018249; doi:10.1016/j.cell.2016.08.005)
Supplement: Document S1. Tables S1–S4 [file mmc1.pdf]

Cell, Volume *166*

Supplemental Information

Tailored Immunogens Direct Affinity Maturation toward HIV Neutralizing Antibodies

Bryan Briney, Devin Sok, Joseph G. Jardine, Daniel W. Kulp, Patrick Skog, Sergey Menis, Ronald Jacak, Oleksandr Kalyuzhniy, Natalia de Val, Fabian Sesterhenn, Khoa M. Le, Alejandra Ramos, Meaghan Jones, Karen L. Saye-Francisco, Tanya R. Blane, Skye Spencer, Erik Georgeson, Xiaozhen Hu, Gabriel Ozorowski, Yumiko Adachi, Michael Kubitz, Anita Sarkar, Ian A. Wilson, Andrew B. Ward, David Nemazee, Dennis R. Burton, and William R. Schief

Table S1. Related to Figure 1. Purpose of GT3 mutations.

Mutation in GT3	Purpose	Mutation in GT6?	Mutation in GT8?
N276D	Eliminate 276 glycan	Y	Y
I277F	Stabilize loop D for VRC01-class binding	Y	Y
T278R	Stabilize loop D; possible VRC01-class contact	Y	Y
F353Y	Improve packing behind loop D	N	Y
N356D	Eliminate glycosylation site introduced by I358T	N	N
I358T	Improve packing on β-sheet; not VRC01 contact	Y	Y
N363P	Eliminate 363 glycan in BG505 that is uncommon in other HIV strains	N#	Y
T372M	Stabilize CD4-binding loop?	N	N
T373N	Add glycosylation site at position 373	N	Y
T455V	Eliminate partially buried and unsatisfied hydroxyl? Possible contact	N	N
S460Y	Stabilize V5? Possible VRC01-class contact	N	Y
T464N	Eliminate the one V5 glycan in BG505	N*	N*

#GT6 lacks a glycan at 363 although it does not use P at that position.
*All V5 glycans have been removed from GT6 and GT8, by other mutations.

Table S2. Related to Figure 1. Antigenic profile of GT3 SOSIP and native-like trimers
Table of monovalent binding affinities as measured by SPR using Fabs as analytes and BG505 SOSIP, BG505 GT3 SOSIP, CD4bs-B SOSIP and CD4bs-C SOSIP as ligands.

	Dissociation constant Kd (nM)												
	**Mat-VRC01**	**GL-VRC01**	**Mat-12A12**	**GL-12A12**	**Mat-CH31**	**GL-CH31**	**Mat-3BNC60**	**Mat-PGV04**	**PGT145[a]**	**PGT151**	**PGT121**	**PGT128**	**35O22**
**BG505 SOSIP**	122	NB	59	NB	137	NB	62	197	6	25*	29	12	276
**BG505 N276D-SOSIP [b]**	41	-	9	-	69	-	11	67	-	234	26	12	30
**GT3 SOSIP**	27*	290	19*	1500	38*	2400*	23*	70*	3.7	39*	40	20	411
**CD4bs-B N276D-SOSIP [b]**	43	-	12	-	811	-	33	165	27	263	19	11	37
**CD4bs-C N276D-SOSIP [b]**	170	-	212	-	3200	-	10000	17000	18	214	20	10	25

* complex kinetics are poor fit to simple 1-to-1 binding model
[a] binding determined using IgG
[b] made in HEK 293S (-GnTI) cells
NB: no binding detected at ≥4μM

Table S3. Related to Figure 3. LCDR1 characteristics for selected VRC01-class bnAbs.

Antibody	VK/VL gene	L-CDR1 sequence*		L-CDR1 length		LCDR1 deletion?
		germline	mature	germline	mature	
12A12	VK1D-33	QDISNY	QGIGSS	6	6	No
12A21	VK1D-33	QDISNY	QGIGSS	6	6	No
3BNC117	VK1D-33	QDISNY	GY	6	2	Yes: 4aa
3BNC60	VK1D-33	QDISNY	GY	6	2	Yes: 4aa
VRC-CH31	VK1D-33	QDISNY	RGIGKD	6	6	No
NIH45-46	VK3-20	QSVSSSY	QSGS	7	4	Yes: 3aa
PGV19	VL2-14	SDVGGYNY	SDFRGFSS	8	8	No
PGV20	VL2-14	SDVGGYNY	STSV	8	4	Yes: 4aa
PGV04	VK3-20	QSVSSSY	SYGH	7	4	Yes: 3aa
VRC01	VK3-20	QSVSSSY	QYGS	7	4	Yes: 3aa
VRC18	VK3-20[a]	QSVSSSY	QGILSNQ	7	7	No
VRC03	VK3-20	QSVSSSY	QGGNA	7	5	Yes: 2aa
VRC06	VK3-20	QSVSSSY	QGGNS	7	5	Yes: 2aa
VRC23	VK3-15[b]	QSVSSN	QGVGSD	6	6	No

*IMGT convention for kappa or lambda chains: L-CDR1 is the stretch between CXXX and XXW.
[a]Georgiev et al., 2013
[b]Zhou et al., 2015

Table S4. Related to Figure 5. Primers used for antibody gene amplification.

Round	Chain	Direction	Sequence
1	Heavy	Forward	GAGGTGCAGCTGCAGGAGTCTGG
1	Heavy	Forward	CAGGTGCAGCTGGTGCAG
1	Heavy	Reverse	AGGGGGCTCTCGCAGGAGACGAGG
1	Heavy	Reverse	GGAAGGTGTGCACACCGCTGGAC
1	Heavy	Reverse	GGAAGGTGTGCACACCACTGGAC
1	Heavy	Reverse	GGAAGGTGTGCACACTGCTGGAC
1	Heavy	Reverse	AGACTGTGCGCACACCGCTGGAC
1	Heavy	Reverse	GAAAGTTCACGGTGGTTATATCC
1	Kappa	Forward	TGCTGCTGCTCTGGGTTCCAG
1	Kappa	Forward	ATTWTCAGCTTCCTGCTAATC
1	Kappa	Forward	TTTTGCTTTTCTGGATTYCAG
1	Kappa	Forward	TCGTGTTKCTSTGGTTGTCTG
1	Kappa	Forward	ATGGAATCACAGRCYCWGGT
1	Kappa	Forward	TCTTGTTGCTCTGGTTYCCAG
1	Kappa	Forward	CAGTTCCTGGGGCTCTTGTTGTTC
1	Kappa	Forward	CTCACTAGCTCTTCTCCTC
1	Kappa	Reverse	GATGGTGGGAAGATGGATACAGTT
1	Lambda	Forward	CAGGCTGTTGTGACTCAG
1	Lambda	Forward	CAACTTGTGCTCACTCAG
1	Lambda	Reverse	GTACCATYTGCCTTCCAGKCCACT
2	Heavy	Forward	CATCCTTTTTCTAGTAGCAACTGCAACCGGTGTACATTCCCAGGTGCAGCTGGTGCAGTCTGG
2	Heavy	Forward	CATCCTTTTTCTAGTAGCAACTGCAACCGGTGTACATTCCGAGGTGCAGCTGCAGGAGTCTGG
2	Heavy	Reverse	GGAAGACCGATGGGCCCTTGGTCGACGCTGAGGAGACGGTGACCGTGG
2	Heavy	Reverse	GGAAGACCGATGGGCCCTTGGTCGACGCTGAGGAGACTGTGAGAGTGG
2	Heavy	Reverse	GGAAGACCGATGGGCCCTTGGTCGACGCTGCAGAGACAGTGACCAGAG
2	Heavy	Reverse	GGAAGACCGATGGGCCCTTGGTCGACGCTGAGGAGACGGTGACTGAGG
2	Heavy	Reverse	GGAAGACCGATGGGCCCTTGGTCGACGCTGAGGAGACGGTGACCAGGG
2	Kappa	Forward	CATCCTTTTTCTAGTAGCAACTGCAACCGGTGTACATTCCGAYATTGTGMTSACMCARWCTMCA
2	Kappa	Reverse	GAAGACAGATGGTGCAGCCACCGTACGTTTGATTTCCAGCTTGGTG
2	Kappa	Reverse	GAAGACAGATGGTGCAGCCACCGTACGTTTTATTTCCAGCTTGGTC
2	Kappa	Reverse	GAAGACAGATGGTGCAGCCACCGTACGTTTTATTTCCAACTTTGTC
2	Kappa	Reverse	GAAGACAGATGGTGCAGCCACCGTACGTTTCAGCTCCAGCTTGGTC
2	Lambda	Forward	CATCCTTTTTCTAGTAGCAACTGCACCGGTTCCTGGGCCCAGGCTGTTGTGACTCAG
2	Lambda	Forward	CATCCTTTTTCTAGTAGCAACTGCACCGGTTCCTGGGCCCAACTTGTGCTCACTCAG
2	Lambda	Reverse	GTTGGCTTGAAGCTCCTCACTCGAGAGGACAGTCAGTTTGGTTCC
2	Lambda	Reverse	GTTGGCTTGAAGCTCCTCACTCGAGAGGACAGTGACCTTGGTTCC
2	Lambda	Reverse	GTTGGCTTGAAGCTCCTCACTCGAGAGGACAGTCAATCTGGTTCC
3	Heavy	Forward	ACACTCTTTCCCTACACGACGCTCTTCCGATCTNNNNGCCAGGGGGAAGACCGATGGGCCCTTGGTCGA
3	Kappa	Forward	ACACTCTTTCCCTACACGACGCTCTTCCGATCTNNNNCGGGAAGATGAAGACAGATGGTGCAGCCACCGT
3	Lambda	Forward	ACACTCTTTCCCTACACGACGCTCTTCCGATCTNNNNGTGGCCTTGTTGGCTTGAAGCTCCTCACTCGA
3	All	Reverse	AGACGTGTGCTCTTCCGATCTXXXXXXXXXXGGTCATGTATCATCCTTTTTCTAGTAGCAACTG
4	All	Forward	CAAGCAGAAGACGGCATACGAGAGATCGGTCTCGGCATTCCTGCTGAAGATXXXXXXXXGTGACTGGAGTT CAGACGTGTGCTCTTCCGATC
4	All	Reverse	AATGATACGGCGACCACCGAGATCTACACTCTTTCCCTACACGACGCTC

Note: Red Xs in the Round 3 reverse primer indicate the position of well-specific barcodes. Blue Xs in the Round 4 reverse primer indicate the position of plate-specific barcodes.
